# Interchangeability of light and virtual microscopy for histopathological evaluation of prostate cancer

**DOI:** 10.1038/s41598-021-82911-z

**Published:** 2021-02-05

**Authors:** Renata Zelic, Francesca Giunchi, Luca Lianas, Cecilia Mascia, Gianluigi Zanetti, Ove Andrén, Jonna Fridfeldt, Jessica Carlsson, Sabina Davidsson, Luca Molinaro, Per Henrik Vincent, Lorenzo Richiardi, Olof Akre, Michelangelo Fiorentino, Andreas Pettersson

**Affiliations:** 1 Clinical Epidemiology Division, Department of Medicine Solna, Karolinska Institutet, Stockholm, Sweden; 2 Department of Pathology, S-Orsola-Malpighi Hospital, Bologna, Italy; 3 Data-Intensive Computing Division, Center for Advanced Studies, Research and Development in Sardinia (CRS4), Pula, Italy; 4 Department of Urology, Faculty of Medicine and Health, Örebro University, Örebro, Sweden; 5 Division of Pathology, A.O. Città Della Salute e Della Scienza Hospital, Turin, Italy; 6 Department of Molecular Medicine and Surgery, Karolinska Institutet, Stockholm, Sweden; 7 Department of Urology, Karolinska University Hospital, Stockholm, Sweden; 8 Cancer Epidemiology Unit, Department of Medical Sciences, University of Turin, and CPO-Piemonte, Turin, Italy; 9 Department of Pathology, Maggiore Hospital, University of Bologna, Bologna, Italy

**Keywords:** Prostate, Prostate cancer, Epidemiology

## Abstract

Virtual microscopy (VM) holds promise to reduce subjectivity as well as intra- and inter-observer variability for the histopathological evaluation of prostate cancer. We evaluated (i) the repeatability (intra-observer agreement) and reproducibility (inter-observer agreement) of the 2014 Gleason grading system and other selected features using standard light microscopy (LM) and an internally developed VM system, and (ii) the interchangeability of LM and VM. Two uro-pathologists reviewed 413 cores from 60 Swedish men diagnosed with non-metastatic prostate cancer 1998–2014. Reviewer 1 performed two reviews using both LM and VM. Reviewer 2 performed one review using both methods. The intra- and inter-observer agreement within and between LM and VM were assessed using Cohen’s kappa and Bland and Altman’s limits of agreement. We found good repeatability and reproducibility for both LM and VM, as well as interchangeability between LM and VM, for primary and secondary Gleason pattern, Gleason Grade Groups, poorly formed glands, cribriform pattern and comedonecrosis but not for the percentage of Gleason pattern 4. Our findings confirm the non-inferiority of VM compared to LM. The repeatability and reproducibility of percentage of Gleason pattern 4 was poor regardless of method used warranting further investigation and improvement before it is used in clinical practice.

## Introduction

The Gleason score is a powerful prognostic factor in prostate cancer^1,2^. Gleason grading is based on subjective histopathological evaluation inevitably leading to inter-observer variability^3–11^. To minimize the inter-observer variability and to reach consensus in controversial areas relating to the Gleason grading system, the International Society of Urological Pathology (ISUP) has performed two major revisions: one in 2005^12^, and one in 2014^13^. In the ISUP 2014 revision, it was, among other things, recommended that cribriform pattern, fused glands and poorly formed glands should be graded as Gleason pattern 4, presence of comedonecrosis and single cells indicates Gleason pattern 5^13^, and that percentage of Gleason pattern 4 should be recorded for all Gleason score 7 cores^14^. The inter-observer agreement for these and other histopathological features has so far been little investigated^15–17^.

Traditionally, the histopathological evaluation has been conducted using light microscopy (LM). Advancements in whole slide imaging and software development have led to the development of digital pathology and virtual microscopy (VM)^18,19^. Although digital pathology and VM are now being introduced in clinical practice, it has hitherto mostly been used for educational purposes, quality assurance, research or for second opinion^20–22^. VM, with or without artificial intelligence features, holds promise to minimize subjectiveness in the slide interpretation, improve the inter-observer agreement, and reduce the review time. Interchangeability of LM and VM has been demonstrated for primary and secondary Gleason pattern, Gleason score, tumour length and perineural invasion^6,23–25^, but not for different Gleason related characteristics (i.e. poorly formed glands, cribriform pattern, comedonecrosis and the percentage of Gleason pattern 4) or for other histopathological characteristics.

As part of an ongoing project, ProMort, which aims at identifying histopathological and molecular markers of lethal prostate cancer^26^, we have developed a new VM system. The present study was conducted to confirm that the study pathologists could use our VM system instead of standard LM for the histopathological review in ProMort. We evaluated the interchangeability of LM and VM by estimating the intra- and inter-method repeatability (i.e., intra-observer agreement) and reproducibility (i.e., inter-observer agreement) for not only the ISUP 2014 Gleason system but also for several less commonly investigated histopathological features.

## Results

In total, 413 cores on 352 slides belonging to 60 cases were reviewed by both reviewers using both LM and VM. Baseline characteristics of the study population are shown in Table 1. Most men were diagnosed prior to 2006 (63%), had Gleason score ≤ 7 (70%), T3 clinical tumor stage (42%), a mean age of 69 years and a mean PSA of 14 ng/ml at diagnosis. The distribution of the characteristics recorded on the core level using LM and VM are reported in Table 2. The distribution of the characteristics recorded on the slide level and the case level summaries are shown in Supplementary Table S1 and S2.

**Table 1:** Baseline characteristics for the 60 study participants.

	N	%
**Year of diagnosis**		
1998–2001	13	21.67
2002–2005	25	41.67
2006–2009	17	28.33
2010–2013	5	8.33
Age at diagnosis (median, IQR)	69 (63.00, 79.00)	
**Clinical tumor stage (T stage)**		
T1c	18	30.00
T2	11	18.33
T3	25	41.67
T4	6	10.00
**Lymph node involvement (N stage)**		
N0	10	16.67
N1	4	6.67
Nx	46	76.67
PSA, ng/ml (median, IQR)	14 (8.10, 28.00)	
**Gleason score**		
4	2	3.51
5	2	3.51
6	21	36.84
3 + 4	4	7.02
4 + 3	11	19.30
8	6	10.53
9	9	15.79
10	2	3.51
Missing	3	
**Primary treatment**		
Conservative	12	20.00
Curative	20	33.33
Androgen deprivation therapy	27	45.00
Death before treatment decision	1	1.67

*N* sample size; *IQR* interquartile range; *PSA* prostate specific antigen.

**Table 2:** Histopathological characteristic evaluated on the core level for the 413 cores which were evaluated by both reviewers using both the light and virtual microscopy.

	Light microscopy			Virtual microscopy		
	Reviewer 1.1	Reviewer 1.2	Reviewer 2	Reviewer 1.1	Reviewer 1.2	Reviewer 2
	N (%)	N (%)	N (%)	N (%)	N (%)	N (%)
**Biopsy core length (mm)**						
Mean (SD)	10.54 (3.73)	10.38 (3.73)	10.38 (3.68)	11.15 (3.95)	11.23 (4.00)	10.81 (3.92)
**Positive core**						
No	155 (37.53)	156 (37.77)	156 (37.77)	156 (37.77)	150 (38.74)	166 (40.19)
Yes	258 (62.47)	257 (62.23)	257 (62.23)	257 (62.23)	253 (61.26)	247 (59.81)
**Tumor length (mm)**						
Mean (SD)	7.59 (4.36)	7.27 (4.33)	7.62 (4.42)	6.85 (4.73)	7.04 (4.71)	6.95 (4.70)
**Primary Gleason pattern**						
3	82 (31.78)	90 (35.02)	79 (30.74)	92 (35.80)	56 (33.60)	89 (36.03)
4	161 (62.40)	165 (64.20)	152 (59.14)	155 (60.31)	158 (62.45)	153 (61.94)
5	15 (5.81)	2 (0.787)	26 (10.12)	10 (3.89)	10 (3.95)	5 (2.02)
**Secondary Gleason pattern**						
3	68 (26.36)	70 (27.24)	64 (24.90)	76 (29.57)	70 (27.67)	67 (27.13)
4	127 (49.22)	131 (50.97)	113 (43.97)	129 (50.19)	119 (47.04)	108 (43.72)
5	63 (24.42)	56 (21.79)	80 (31.13)	52 (20.23)	64 (25.30)	72 (29.15)
**Gleason score**						
6	47 (18.22)	56 (21.79)	45 (17.51)	58 (22.57)	50 (19.76)	55 (22.27)
7	56 (21.71)	48 (18.68)	53 (20.62)	52 (20.23)	55 (21.74)	46 (18.62)
8	82 (31.78)	95 (36.96)	61 (23.74)	85 (33.07)	78 (30.83)	69 (27.94)
9	68 (26.36)	58 (22.57)	90 (35.02)	62 (24.12)	66 (26.09)	77 (31.17)
10	5 (1.94)	–	8 (3.11)		4 (1.58)	
**Gleason Grade groups**						
1	47 (18.22)	56 (21.79)	45 (17.51)	58 (22.57)	50 (19.76)	55 (22.27)
2	35 (13.57)	34 (13.23)	34 (13.23)	34 (13.23)	35 (13.83)	34 (13.77)
3	21 (8.14)	14 (5.45)	19 (7.39)	18 (7.00)	20 (7.91)	12 (4.86)
4	82 (31.78)	95 (36.96)	61 (23.74)	85 (33.07)	78 (30.83)	69 (27.94)
5	73 (28.29)	58 (22.57)	98 (38.13)	62 (24.12)	70 (27.67)	77 (31.17)
**Percentage of Gleason pattern 4**						
Mean (SD)	43.75 (29.16)	35.94 (24.90)	42.92 (25.37)	29.63 (22.32)	31.46 (22.35)	26.07 (18.28)
**Perineural invasion**						
No	200 (77.52)	218 (84.82)	198 (77.04)	213 (83.53)	205 (81.35)	209 (84.96)
Yes	58 (22.48)	39 (15.18)	59 (22.96)	42 (16.47)	47 (18.65)	37 (15.04)
Missing	–	–	–	2	1	1
**Intraductal carcinoma**						
No	256 (99.22)	252 (98.05)	243 (94.55)	248 (97.25)	248 (98.41)	241 (97.97)
Yes	2 (0.78)	5 (1.95)	14 (5.45)	7 (2.75)	4 (1.59)	5 (2.03)
Missing	–	–	–	2	1	1
**Ductal carcinoma**						
No	258 (100.00)	257 (100.00)	256 (99.61)	255 (100.00)	252 (100.00)	246 (100.00)
Yes	0	0	1 (0. 39)	0	0	0
Missing	–	–	–	2	1	1
**Poorly formed glands**						
No	97 (38.13)	78 (32.28)	102 (40.49)	77 (30.20)	72 (28.57)	96 (39.02)
Yes	161 (61.87)	179 (67.72)	155 (59.51)	178 (69.80)	180 (71.43)	150 (60.98)
Missing	–	–	–	2	1	1
**Cribriform pattern**						
No	145 (56.20)	155 (60.31)	130 (50.58)	153 (60.00)	151 (59.92)	148 (60.16)
Yes	113 (43.80)	102 (39.69)	127 (49.42)	102 (40.00)	101 (40.08)	98 (39.84)
Missing	–	–	–	2	1	1
**Hypernephroid pattern**						
No	254 (98.45)	257 (100.00)	248 (96.50)	255 (100.00)	252 (100.00)	246 (100.00)
Yes	4 (1.55)	0	9 (3.50)	0	0	0
Missing	–	–	–	2	1	1
**Mucinous carcinoma**						
No	252 (97.67)	253 (98.83)	243 (94.55)	251 (98.43)	247 (98.02)	243 (98.78)
Yes	6 (2.33)	3 (1.17)	14 (5.45)	4 (1.57)	5 (1.98)	1.22
Missing	–	1	–	2	1	1
**Comedonecrosis**						
No	246 (95.35)	247 (96.11)	233 (90.66)	242 (94.90)	238 (94.47)	233 (94.72)
Yes	12 (4.65)	10 (3.89)	24 (9.34)	13 (5.08)	14 (5.53)	13 (5.28)
Missing	–	–	–	2	1	1

*N* sample size; *SD* standard deviation; Reviewer 1.1, First review by Reviewer 1; Reviewer 1.2, Second review by Reviewer 1 .

### Repeatability

#### Intra-method, intra-observer agreement

**Figure 1:**
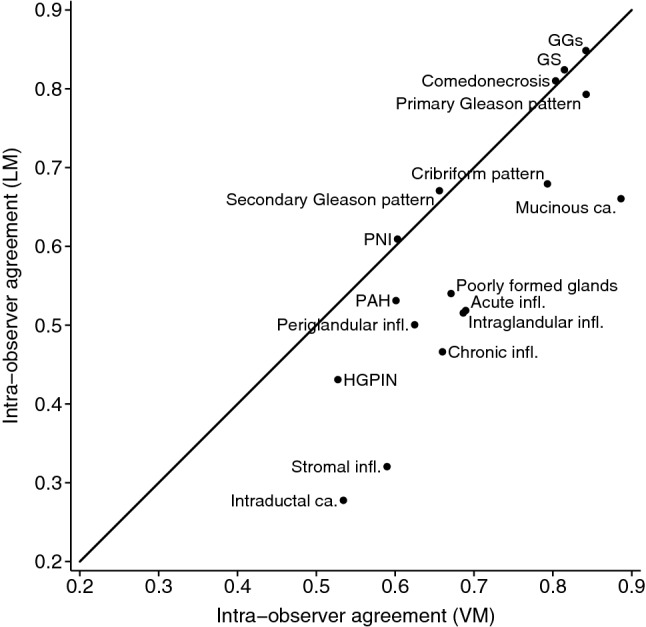
Repeatability plot for all characteristics evaluated on the core and slide level. Note: Intra-observer agreement refers to the agreement of the Reviewer 1.1 vs. Reviewer 1.2. *LM* light microscopy; *VM* virtual microscopy; *GGs* Gleason Grade Groups; *GS* Gleason score; *PNI* perineural invasion; *PAH* postatrophic hyperplasia; *HGPIN* high-grade prostatic intraepithelial neoplasia; *infl* Inflammation; *ca* carcinoma.

For both the core length and tumour length, the limits of agreement were narrower for VM than for LM, indicating better repeatability using VM (Supplementary Figs. S1 and S2). The agreement for the Gleason related characteristics was similar for LM vs. VM (Fig. 1), ranging from substantial to almost perfect (primary Gleason pattern: κ_wLM_ = 0.79 vs. κ_wVM_ = 0.84; secondary Gleason pattern: κ_wLM_ = 0.67 vs. κ_wVM_ = 0.66; GGs: κ_wLM_ = 0.85 vs. κ_wVM_ = 0.84) (Supplementary Fig. S3). The agreement for comedonecrosis and perineural invasion was similar using the two methods, while it was higher on VM vs. LM for cribriform pattern, poorly formed glands, mucinous and intraductal carcinoma and for all slide level characteristics (Supplementary Figs. S3 and S4), indicating either comparable or better repeatability on VM vs. LM (Fig. 1). The agreement for the percentage of Gleason pattern 4 was overall poor, but somewhat better on VM vs. LM in terms of average differences and width of limits of agreement (Table 3, Supplementary Fig. S5).

**Table 3:** Intra- and inter-method, intra- and inter-observer agreement for the percentage of Gleason pattern 4, evaluated on the core level.

	N	Difference					Limits of agreement
		Mean	SD	Median	Min	Max	
**Intra-method agreement**							
Intra-observer agreement							
Light 1.1 vs. Light 1.2	40	10.23	27.30	10	− 60	80	− 43.29, 63.74
Virtual 1.1 vs. Virtual 1.2	42	− 2.99	17.72	− 0.13	− 56.71	28.24	− 37.71, 31.74
Inter-observer agreement							
Light 1.1 vs. Light 2	47	− 3	20.05	0	− 80	40	− 42.29, 36.29
Light 1.2 vs. Light 2	36	− 6.53	24.84	− 2.5	− 70	60	− 55.21, 42.15
Virtual 1.1 vs. Virtual 2	37	1.20	19.40	3.49	− 48.95	38.82	− 36.82, 39.23
Virtual 1.2 vs. Virtual 2	37	4.82	22.68	3.55	− 50.06	51.26	− 39.63, 49.27
**Inter-method agreement**							
Intra-observer agreement							
Light 1.1 vs. Virtual 1.1	45	18.61	21.70	19.43	− 51.47	61.51	− 23.92, 61.14
Light 1.1 vs. Virtual 1.2	43	18.11	21.34	15.17	− 21.66	62.17	− 23.71, 59.94
Light 1.2 vs. Virtual 1.1	38	8.64	23.99	11.33	− 63.45	70	− 38.38, 55.65
Light 1.2 vs. Virtual 1.2	40	3.14	26.61	7.48	− 70.18	62.33	− 49.01, 55.29
Light 2 vs. Virtual 2	35	21.73	25.57	15.51	− 34.90	78.03	− 28.39, 71.85
Inter-observer agreement							
Light 1.1 vs. Virtual 2	39	21.05	26.27	12.71	− 34.90	76.36	− 30.43, 72.54
Light 1.2 vs. Virtual 2	41	11.97	19.06	10.00	− 34.90	59.4	− 25.38, 49.33
Light 2 vs. Virtual 1.1	41	19.38	22.37	22.37	− 54.47	59.38	− 24.46, 63.21
Light 2 vs. Virtual 1.2	40	18.10	19.92	18.18	− 29.58	60.20	− 20.95, 57.15

*N* sample size; *SD* standard deviation; Light 1.1, First review by Reviewer 1 on light microscopy; Light 1.2, Second review by Reviewer 1 on light microscopy; Virtual 1.1, First review by Reviewer 1 on virtual microscopy; Virtual 1.2, Second review by Reviewer 1 on virtual microscopy; Light 2, Reviewer 2 on light microscopy; Virtual 2, Reviewer 2 on virtual microscopy.

### Reproducibility

#### Intra-method, inter-observer agreement

**Figure 2:**
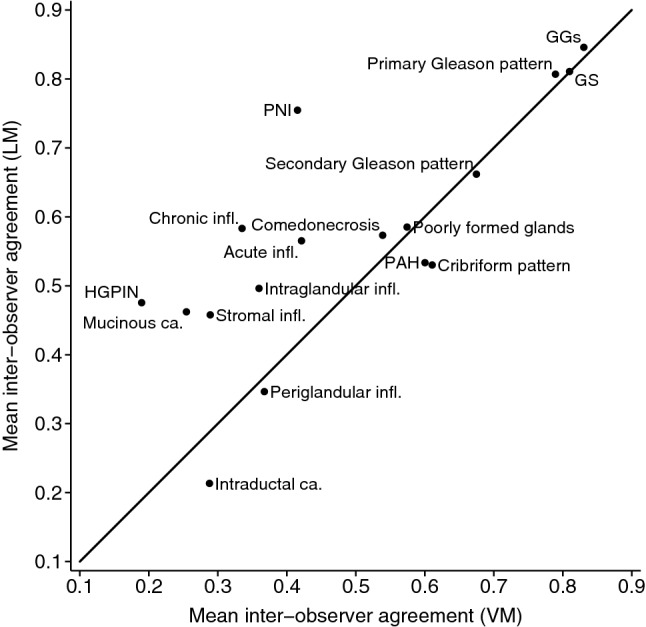
Reproducibility plot for all characteristics evaluated on the core and slide level. Note: Mean inter-observer agreement refers to the mean of the agreements between the Reviewer 1.1 vs. Reviewer 2 and Reviewer 1.2. vs. Reviewer 2. *LM* light microscopy; *VM* virtual microscopy; *GGs* Gleason Grade Groups; *GS* Gleason score; *PNI* perineural invasion; *PAH* Postatrophic hyperplasia; *HGPIN* High-grade prostatic intraepithelial neoplasia; *infl* Inflammation; *ca* carcinoma.

For both the core length and tumour length, the average inter-observer difference was close to zero for both LM and VM, with narrower limits of agreement for VM (Supplementary Figs. S6 and S7), indicating good reproducibility using both methods. The agreement for the Gleason related characteristics was similar for the two methods (Fig. 2), ranging from moderate/substantial to almost perfect (primary Gleason pattern: κ_wLM_ = 0.72–0.89 vs. κ_wVM_ = 0.78–0.80; secondary Gleason pattern: κ_wLM_ = 0.58–0.74 vs. κ_wVM_ = 0.67–0.68; GGs: κ_wLM_ = 0.80–0.89 vs. κ_wVM_ = 0.83) (Supplementary Fig. S8), indicating good reproducibility using both methods. The agreement for the remaining characteristics was similar for the two methods, except for mucinous carcinoma, perineural invasion, high-grade prostatic intraepithelial neoplasia (HGPIN) and chronic inflammation where it was higher on LM (Fig. 2, Supplementary Figs. S8 and S9), indicating better reproducibility using LM. The agreement for the percentage of Gleason pattern 4 was overall poor, with average differences close to 0 for both methods but with wide limits of agreement (Table 3, Supplementary Fig. S10).

### Interchangeability

#### Inter-method, intra-observer agreement

**Figure 3:**
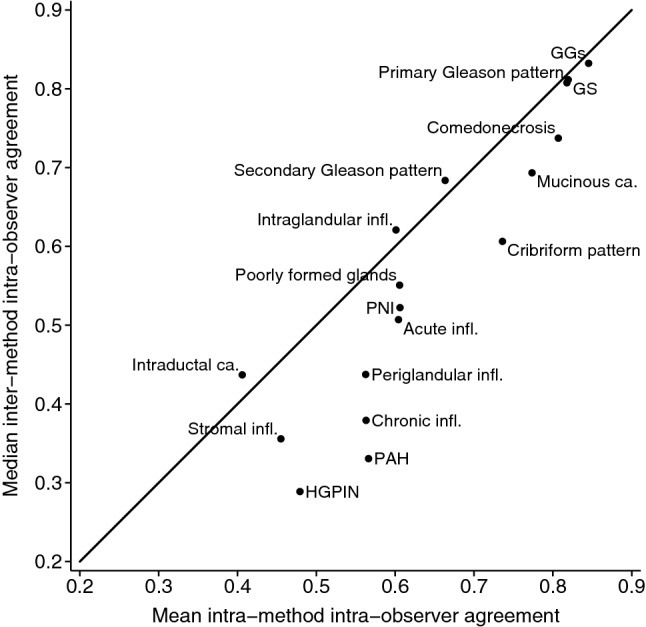
Interchangeability plot for all characteristics evaluated on the core and slide level (median inter-method intra-observer agreement vs. mean intra-method intra-observer agreement). Note: Median inter-method intra-observer agreement refers to the median of the agreements between the Reviewer 1.1 on LM vs. VM, Reviewer 1.2 on LM vs. VM and Reviewer 2 on LM vs. VM. Mean intra-method intra-observer agreement refers to the mean of the agreements between the Reviewer 1.1 vs. Reviewer 1.2 on LM and on VM. LM, light microscopy; *VM* virtual microscopy; *GGs* Gleason Grade Groups; GS, Gleason score; *PNI* Perineural invasion; *PAH* Postatrophic hyperplasia; *HGPIN* High-grade prostatic intraepithelial neoplasia; *infl* Inflammation; *ca* carcinoma.

The core length was on average 1 mm shorter, while the tumour length was 1 mm longer, when measured by the same reviewer using VM vs. LM, with similar limits of agreement for all intra-observer comparisons (Supplementary Figs. S11 and S12). The median inter-method intra-observer agreement for the Gleason related characteristics was similar to the average intra-method intra-observer agreement, indicating interchangeability of LM and VM (Fig. 3). It ranged from moderate to almost perfect (primary Gleason pattern: κ_w_ = 0.69–0.88; secondary Gleason pattern: κ_w_ = 0.59–0.75; GGs: κ_w_ = 0.81–0.87) (Supplementary Fig. S13). Similarly, the median inter-method intra-observer agreement for the remaining core level characteristics was similar to the average intra-method, intra-observer agreement, indicating interchangeability of LM and VM (Fig. 3, Supplementary Fig. S13). However, for most of the slide level characteristics, median inter-method intra-observer agreement was lower than the average intra-method intra-observer agreement (Fig. 3, Supplementary Fig. S14), probably due to the higher intra-observer agreement on VM vs. LM. On average, the percentage of Gleason pattern 4 measured using LM was 3.14–21.73 percentage points larger then when using VM, with wide limits of agreement (Table 3, Supplementary Fig. S15).

#### Inter-method, inter-observer agreement

**Figure 4:**
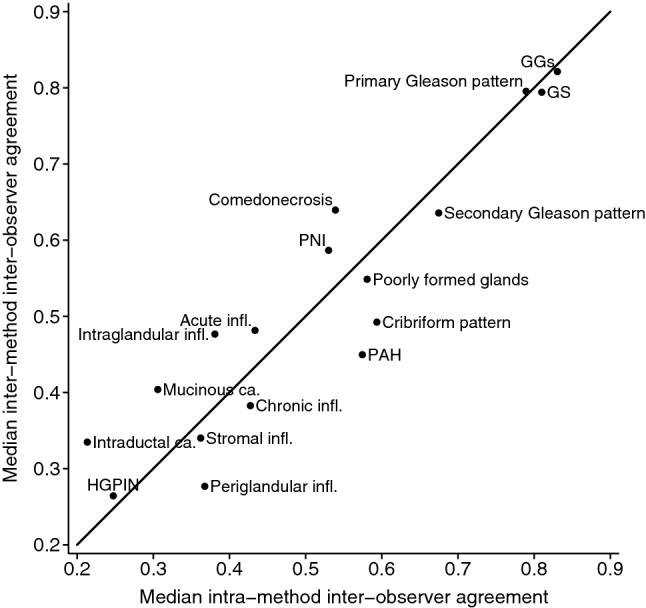
Interchangeability plot for all characteristics evaluated on the core and slide level. Note: Median intra-method inter-observer agreement refers to the median of the agreements between the Reviewer 1.1 vs. Reviewer 2 and Reviewer 1.2 vs. Reviewer 2 on LM and on VM. Median inter-method inter-observer agreement refers to the median of the agreements between the Reviewer 1.1 on LM vs. Reviewer 2 on VM, Reviewer 2 on LM vs. Reviewer 1.1 on VM, Reviewer 2 on LM vs. Reviewer 1.2 on VM and Reviewer 1.2 on LM vs. Reviewer 2 on VM. LM, light microscopy; *VM* virtual microscopy; *GGs* Gleason Grade Groups; *GS* Gleason score; *PNI* Perineural invasion; *PAH* Postatrophic hyperplasia; *HGPIN* High-grade prostatic intraepithelial neoplasia; *infl* Inflammation; *ca* carcinoma.

The inter-observer agreement was on average 1 mm shorter for core length, and 1 mm longer for tumour length, when measured by the two reviewers using LM vs. VM (Supplementary Figs. S16 and S17). The median inter-method inter-observer agreement for the Gleason related characteristics was similar to the median intra-method, inter-observer agreement, indicating interchangeability of VM and LM (Fig. 4). It ranged from moderate to almost perfect (primary Gleason pattern: κ_w_ = 0.76–0.88; secondary Gleason pattern: κ_w_ = 0.53–0.77; GGs: κ_w_ = 0.81–0.88) (Supplementary Fig. S18). Similarly, median inter-method inter-observer agreement for the remaining characteristics was similar to the median intra-method inter-observer agreement, indicating interchangeability of VM and LM (Fig. 4, Supplementary Figs. S18 and S19). The average difference in the percentage of Gleason pattern 4 measured using LM vs. VM was 11.97–21.05 percentage points, with very wide limits of agreement (Table 3, Supplementary Fig. S20).

### Case level results

Overall, the case level results were similar to the core level results (Supplementary Figs. S21–S32).

## Discussion

This is the first study with detailed, replicate reviews of the ISUP 2014 Gleason grading system on both LM and VM. We found interchangeability of LM and VM, adding further to the body of evidence showing non-inferiority of VM compared to LM. Furthermore, on both LM and VM, we found good repeatability and reproducibility for all the evaluated histopathological characteristics, except for the percentage of Gleason pattern 4, presence of inflammation, HGPIN and postatrophic hyperplasia (PAH). The repeatability and reproducibility for these features should be investigated further and improved before they are used in clinical practice.

In line with previous studies, our study shows that VM and LM can be used interchangeably for Gleason grading, core length, tumour length and perineural invasion. Previous studies have reported moderate to almost perfect inter-method, intra-observer agreement, and moderate to substantial intra-method, inter-observer agreement, for both LM and VM for primary and secondary Gleason pattern, Gleason score, tumour length and perineural invasion^6,23–25^. We additionally report similar intra-method, intra-observer agreement for primary and secondary Gleason pattern, Gleason score and perineural invasion, as well as better agreement for core length and tumour length on VM vs. LM. Our study is unique in that we also evaluated the interchangeability of VM and LM for cribriform pattern, poorly formed glands, comedonecrosis, percentage of Gleason pattern 4, and intraductal and mucinous carcinoma, inflammation, HGPIN and PAH. Even when we did not find good repeatability and/or reproducibility (i.e. for percentage of Gleason pattern 4, inflammation, HGPIN and PAH), our findings were similar on LM and VM, indicating that also for these characteristics LM and VM can be used interchangeably.

In prior studies, Gleason grading reproducibility has typically been evaluated by assessing the inter-observer agreement on diagnostic biopsies using LM. The reported agreement varies from no to almost perfect for primary and secondary Gleason pattern, Gleason score and GGs among general pathologists^3–5,7–9,27–29^, and, as in our study, from moderate to substantial for Gleason score among uropathologists^3,10,11^. Compared to previous studies, we observed somewhat better inter-observer agreement for secondary Gleason pattern and the GGs, which could be explained by Reviewer 1 and Reviewer 2 having worked together for more than 7 years. The intra-observer agreement has been less studied, with few reports showing slightly lower agreement for primary and secondary Gleason pattern, and Gleason score on LM than in our study^3,29^. The differences between our study and previous studies for both the inter- and intra-observer agreement may be due to the use of different statistical methods to evaluate agreement, or because many of the previous studies were performed either before the ISUP 2005 revision or shortly thereafter.

We found moderate to substantial intra- and inter-observer agreement both within and between LM and VM for poorly formed glands, cribriform pattern and comedonecrosis, indicating good repeatability, reproducibility and interchangeability of LM and VM for these features. Previous studies report no to substantial inter-observer agreement for “poorly formed glands”^15,16^, poor to substantial agreement for cribriform pattern^15,16^, and moderate intra-observer agreement for comedonecrosis^17^. In the ISUP 2014 revision it was recommended that the percentage of Gleason pattern 4 should be reported for cores with Gleason score 7^14^, and the reported inter-observer agreement using LM is moderate to substantial^16^. However, a previous study reported that the average percentage of Gleason pattern 4 evaluated using LM is almost twice as large as the VM estimate^30^, indicating overestimation of the percentage of Gleason pattern 4 on LM. We also found higher average percentage of Gleason pattern 4 on LM vs. VM. The intra-observer agreement in our study was somewhat better on VM compared to LM. The wide limits of agreement, however, indicate either a poor repeatability and reproducibility on both methods or a strong influence of outliers given a small sample size used in this analysis. Taken together, previous data and our data suggest that the repeatability and reproducibility for percentage Gleason pattern 4 should be further investigated and improved before it is used in clinical practice.

To the best of our knowledge, no previous study has evaluated the agreement for intraductal carcinoma, mucinous carcinoma, presence of inflammation, HGPIN and PAH in prostate cancer biopsy samples. We found moderate to substantial intra- and inter-method, intra-observer agreement for intraductal and mucinous carcinoma, except for Reviewer 2. The intra-method, inter-observer agreement, however, was not as good, especially when VM was used. Given that Reviewer 1 had more experience with VM, the inter-method, intra-observer and intra-method, inter-observer agreement could potentially be improved by additional VM training for Reviewer 2. Our results also indicate moderate reproducibility for the presence of inflammation, HGPIN and PAH, regardless of the method used. The repeatability, however, was better when VM was used. Since all of these features are not commonly reported and/or are rare, the guidelines for their assignment are not as uniform as for e.g. Gleason grading^31,32^. Thus, consensus on their assignment could further improve both repeatability and reproducibility.

The key limitation of this study is that we evaluated agreement only between two uropathologists that have also worked together, which may partly explain the generally high inter-observer agreement we observed for several key characteristics. Our findings, thus, may not reflect the agreement between general or unrelated pathologists. However, this does not affect our key finding of interchangeability of LM and VM as we found that the inter-observer, as well as intra-observer, agreement was similar on LM vs. VM, and they were both similar to the inter-method agreement. Furthermore, it is likely that unequal training in the use of VM, as well as use of small 12.9-inch display on the 2018 iPad Pro, which was used to run VM, could preclude the identification of small (e.g., smaller cancer foci) or rare features (e.g., intraductal or mucinous carcinoma). This could explain why Reviewer 2 identified less cores with cancer on VM compared with LM. As we did not have information on consensus diagnosis, we could not evaluate whether more cores with cancer are missed using LM or VM. However, at least one previous study reported that compared with the consensus diagnosis, more pathologists missed invasive cancers on LM than on VM, and the inter-observer agreement on invasive cancer was better on VM vs. LM^24^. Given the small number of slides evaluated in this study (n = 8), this important aspect should be further investigated.

## Conclusion

Our study confirms that VM and LM can be used interchangeably. In addition, we found good repeatability for primary and secondary Gleason pattern, Gleason score, GGs and perineural invasion as well as for the presence of poorly formed glands, cribriform pattern and comedonecrosis. The repeatability and/or reproducibility for the percentage of Gleason pattern 4 and other less commonly reported features was, however, poor on both LM and VM, indicating a lack of a consensus and/or pathologists’ training in assignment of these features. Emphasis should be put on improving the repeatability and reproducibility of these features before they are used in clinical practice.

## Materials and methods

### Study sample

ProMort is a case–control study nested in the National Prostate Cancer Register of Sweden (NPCR), a clinical cancer registry containing data on virtually all men in Sweden diagnosed with prostate cancer since 1998^33^. In this study, we included a random sample of cases/controls from ProMort (n = 60) diagnosed with non-metastatic prostate cancer (i.e., non-M1) between January 1, 1998 and December 31, 2014 in two out of Sweden’s 21 counties (n = 25 from Örebro county, and n = 35 from Värmland county). The regional Swedish Ethics Review Authority (Etikprövningsmyndigheten) in Stockholm, Sweden approved this study (reference number: 2017/1705-32) and the requirement for informed consent was waived. All analyses were performed in accordance with relevant guidelines and regulations.

### Slide digitalization and managing

The diagnostic biopsy slides from the 60 study members were retrieved from the Pathology wards at the Örebro University Hospital, Örebro, Sweden, and Karlstad Central Hospital, Karlstad, Sweden, and scanned at Örebro University Hospital using the Pannoramic 250 Flash II digital slide scanner (3DHistech Ltd., Budapest, Hungary) with a 40 × objective, yielding images with a resolution of 0.19 microns/pixel. The original slide labels were replaced with a new study identifier (study ID).

After scanning, the images were uploaded to a VM system developed by the Centre for Advanced Studies, Research and Development in Sardinia (CRS4), Pula, Italy. The technical details of the VM system will be published in a separate article (under preparation) and a more detailed description is provided in the Supplementary methods. In short, the VM system is composed of two integrated components: (1) ome_seadragon^34^, a plugin for the Open Microscopy Environment Remote Objects (OMERO) platform^35^ which enables viewing, handling and annotation of the 3DHistech images, and (2) the ProMort Image Management System (https://github.com/crs4/ProMort), a clinical annotation platform which manages the review worklist and the clinical annotation process. The ome_seadragon plugin adds Deep Zoom Image format support to the OMERO platform and enables interactive mark-up of regions of interest (ROIs) on the slide and automated measurements of marked ROIs (e.g., length or area of the ROI). The ProMort Image Management System embeds an ome_seadragon client allowing for user-friendly navigation and clinical annotation of digitalized slides using a dedicated user interface specifically designed for ProMort.

Both the ome_seadragon client and the ProMort Image Management System are web-based applications developed to run on all modern browsers and require no specific hardware or operative system. For this study, the pathologists used either a desktop PC, with a 22 inch Olivetti OLISCREEN22 display, running Google Chrome browser or a 2018 iPad Pro, with a 12.9 inch display, running Safari browser.

### Histopathological review

The histopathological review was performed according to a pre-specified protocol. To avoid the use of the same study ID in different reviews, a new random identifier was automatically assigned to each slide by the ProMort platform worklist manager. The link between this new random ID and the study ID was known only to the ProMort worklist manager.

Two genitourinary pathologists (F.G., M.F.) performed the histopathological review according to the WHO classification of tumours of the urinary system and male genital organs issued in 2016^36^, following a pre-specified protocol, with a minimum washout period of 2 weeks between each review. The first pathologist (Reviewer 1), with 11 years of experience (6 years as a dedicated genitourinary pathologist), performed two reviews using LM and two reviews using VM, while the second pathologist (Reviewer 2), with 27 years of experience (13 years as a dedicated genitourinary pathologist), performed one review using LM and one review using VM. This approach allowed us to estimate both the intra- and inter-observer agreement for both LM and VM. Both pathologists had been consulted during development of the VM system and were familiar with its functionality. Both pathologists were blinded to the original clinical and histopathological information of all slides.

For both LM and VM, the histopathological review started with a quality control of all diagnostic slides. We excluded slides that lacked tissue or for quality reasons (Supplementary Table S3). For each core, we recorded core length (mm) and presence of cancer. For each core with cancer, we recorded tumour length (mm), primary and secondary Gleason pattern, Gleason pattern related characteristics (i.e., poorly formed glands, cribriform pattern, comedonecrosis), Gleason Grade Groups (GGs), and presence and absence of perineural invasion, intraductal, ductal, hypernephroid and mucinous carcinoma. For cores with Gleason score 7, we measured the percentage of Gleason pattern 4 on LM by “eye-balling” (categorized as < 10%, 10–19%, 20–29% etc.^14^) and on VM as the area of Gleason pattern 4 divided by the total tumour area. For each slide (but not for each core), we recorded information on the presence or absence of acute, chronic, periglandular, intraglandular and stromal inflammation, HGPIN and PAH.

### Statistical analyses

We assessed the intra- and inter-observer agreement within each method and between the two methods using Cohen’s kappa (κ) for binary variables^37^, weighted Cohen’s kappa (κ_w_) with linear weights for ordinal variables^38^, and Bland and Altman’s limits of agreement for continuous variables^39^. For descriptive purposes, κ/κ_w_ < 0 was considered as no agreement, 0–0.20 slight, 0.21–0.40 fair, 0.41–0.60 moderate, 0.61–0.80 substantial, and 0.81–1 almost perfect agreement^40^.

Agreement was evaluated on the core level for all characteristics recorded on the core level and on the slide level for characteristics recorded only on the slide level. For characteristics typically reported on the case level (i.e., the primary and secondary Gleason pattern, Gleason score, GGs, total core length, total tumour length, and perineural invasion), we also evaluated the case level agreement.

Analyses were conducted in Stata (version 12.1, StataCorp, College Station, Texas, USA).

## Supplementary Information

Supplementary Information.
