# Supplementary material for: Interchangeability of light and virtual microscopy for histopathological evaluation of prostate cancer
Source: Sci Rep. 2021 Feb 5;11:3257. doi: 10.1038/s41598-021-82911-z (PMC7864946; doi:10.1038/s41598-021-82911-z)

Supplementary Materials for

Interchangeability of light and virtual microscopy for histopathological evaluation of prostate cancer

Renata Zelic, Francesca Giunchi, Luca Lianas, Cecilia Mascia, Gianluigi Zanetti, Ove Andrén, Jonna Fridfeldt, Jessica Carlsson, Sabina Davidsson, Luca Molinaro, Per Henrik Vincent, Lorenzo Richiardi, Olof Akre, Michelangelo Fiorentino, Andreas Pettersson

Correspondence to: renata.zelic@ki.se

Conflict of Interest: None to declare.

Table of Contents:

Supplementary methods 4

Supplementary Table S1. Histopathological characteristics evaluated on the slide level for 352 slides which were evaluated by both reviewers using both the light and virtual microscopy ... 6

Supplementary Table S2. Case-level summary of histopathological characteristics for 60 cases 7

Supplementary Table S3. Reasons for exclusion of diagnostic slides 8

Supplementary Figure S1. Bland and Altman plot of the intra-method intra-observer agreement for biopsy core length measured in millimeters, evaluated on the core level........... 9

Supplementary Figure S2. Bland and Altman plot of the intra-method intra-observer agreement for length of tumor in the biopsy core measured in millimeters, evaluated on the core level 10

Supplementary Figure S3. Intra-method intra-observer agreement for characteristics evaluated on the core level 11

Supplementary Figure S4. Intra-method intra-observer agreement for characteristics evaluated on the slide level... 12

Supplementary Figure S5. Bland and Altman plot of the intra-method intra-observer agreement for the percentage of Gleason pattern 4, evaluated on the core level..................... 13

Supplementary Figure S6. Bland and Altman plot of the intra-method inter-observer agreement for biopsy core length measured in millimeters, evaluated on the core level......... 14

Supplementary Figure S7. Bland and Altman plot of the intra-method inter-observer agreement for the length of tumor in the biopsy core measured in millimeters, evaluated on the core level 15

Supplementary Figure S8. Intra-method inter-observer agreement for characteristics evaluated on the core level 16

Supplementary Figure S9. Intra-method inter-observer agreement for characteristics evaluated on the slide level... 17

Supplementary Figure S10. Bland and Altman plot of the intra-method inter-observer agreement for the percentage of Gleason pattern 4, evaluated on the core level..................... 18

Supplementary Figure S11. Bland and Altman plot of the inter-method intra-observer agreement for the biopsy core length measured in millimeters, evaluated on the core level... 19

Supplementary Figure S12. Bland and Altman plot of the inter-method intra-observer agreement for the length of tumor in the biopsy core measured in millimeters, evaluated on the core level 20

Supplementary Figure S13. Inter-method intra-observer agreement for characteristics evaluated on the core level 21

Supplementary Figure S14. Inter-method intra-observer agreement for characteristics evaluated on the slide level 22

Supplementary Figure S15. Bland and Altman plot of the inter-method intra-observer agreement for the percentage of Gleason pattern 4, evaluated on the core level 23

Supplementary Figure S16. Bland and Altman plot of the inter-method inter-observer agreement for the biopsy core length measured in millimeters, evaluated on the core level... 24

Supplementary Figure S17. Bland and Altman plot of the inter-method inter-observer agreement for the length of tumor in the biopsy core measured in millimeters, evaluated on the core level 25

Supplementary Figure S18. Inter-method inter-observer agreement for characteristics evaluated on the core level 26

Supplementary Figure S19. Inter-method inter-observer agreement for characteristics evaluated on the slide level 27

Supplementary Figure S20. Bland and Altman plot of the inter-method inter-observer agreement for the percentage of Gleason pattern 4, evaluated on the core level 28

Supplementary Figure S21. Bland and Altman plot of the intra-method intra-observer agreement for the total biopsy length measured in millimeters, evaluated on the case level .. 29

Supplementary Figure S22. Bland and Altman plot of the intra-method intra-observer agreement for the total length of tumor in the biopsy measured in millimeters, evaluated on the case level 30

Supplementary Figure S23. Intra-method intra-observer agreement evaluated on the case level 31

Supplementary Figure S24. Bland and Altman plot of the intra-method inter-observer agreement for the total biopsy length measured in millimeters, evaluated on the case level .. 32

Supplementary Figure S25. Bland and Altman plot of the intra-method inter-observer agreement for the total length of tumor in the biopsy measured in millimeters evaluated on the case level ... 33

Supplementary Figure S26. Intra-method inter-observer agreement evaluated on the case level ... 34

Supplementary Figure S27. Bland and Altman plot of the inter-method intra-observer agreement for the total biopsy length measured in millimeters, evaluated on the case level ... 35

Supplementary Figure S28. Bland and Altman plot of the inter-method intra-observer agreement for the total length of tumor in the biopsy measured in millimeters, evaluated on the case level ... 36

Supplementary Figure S29. Inter-method intra-observer agreement evaluated on the case level ... 37

Supplementary Figure S30. Bland and Altman plot of the inter-method inter-observer agreement for the total biopsy length measured in millimeters, evaluated on the case level ... 38

Supplementary Figure S31. Bland and Altman plot of the inter-method inter-observer agreement for the total length of tumor in the biopsy measured in millimeters, evaluated on the case level ... 39

Supplementary Figure S32. Inter-method inter-observer agreement evaluated on the case level ... 40

Supplementary methods

The virtual microscopy system

The virtual microscopy system was developed by Centre for Advanced Studies, Research and Development in Sardinia (CRS4, www.crs4.it) and it comprises two integrated components (Figure 1):

1. ome_seadragon (https://github.com/crs4/ome_seadragon), a plugin for the Open Microscopy Environment Remote Objects (OMERO) platform,[1] which enables viewing, handling and annotation of the 3DHistech images. The image management is based on the OMERO.server which supports over 140 different image formats and allows for storing of meta-information (e.g., classification TAGs or Regions of Interests (ROIs)). The ome_seadragon simplifies the integration of the images stored within OMERO into external web systems,[2] adds Deep Zoom Image format (DZI) support to OMERO and, through OpenSlide libraries (https://openslide.org),[3] increases the number of supported image formats. The user side of ome_seadragon is a specialized viewer based on the open source viewer OpenSeadragon (https://openseadragon.github.io). This viewer acts as a Virtual Microscope and can be easily embedded into external web services. Real time annotation tools which are based on Paper.js libraries (http://paperjs.org) enable navigation on a digital slide and annotation by drawing different 2D shapes, as well as taking precise measures (e.g., ROI length or area).
2. The ProMort Image Management System (https://github.com/crs4/ProMort), a web application developed by the CRS4 to manage the review worklist and clinical annotation process related to the ProMort study. The clinical annotation comprises two main steps: the definition of the ROIs and the clinical annotation of the ROIs. By embedding the ome_seadragon viewer (i.e., the Virtual Microscope), the ProMort Image Management System allows users to navigate and annotate digital slides while creating new ROIs. Clinical annotations are performed via a dedicated user interface which has been designed specifically for ProMort.

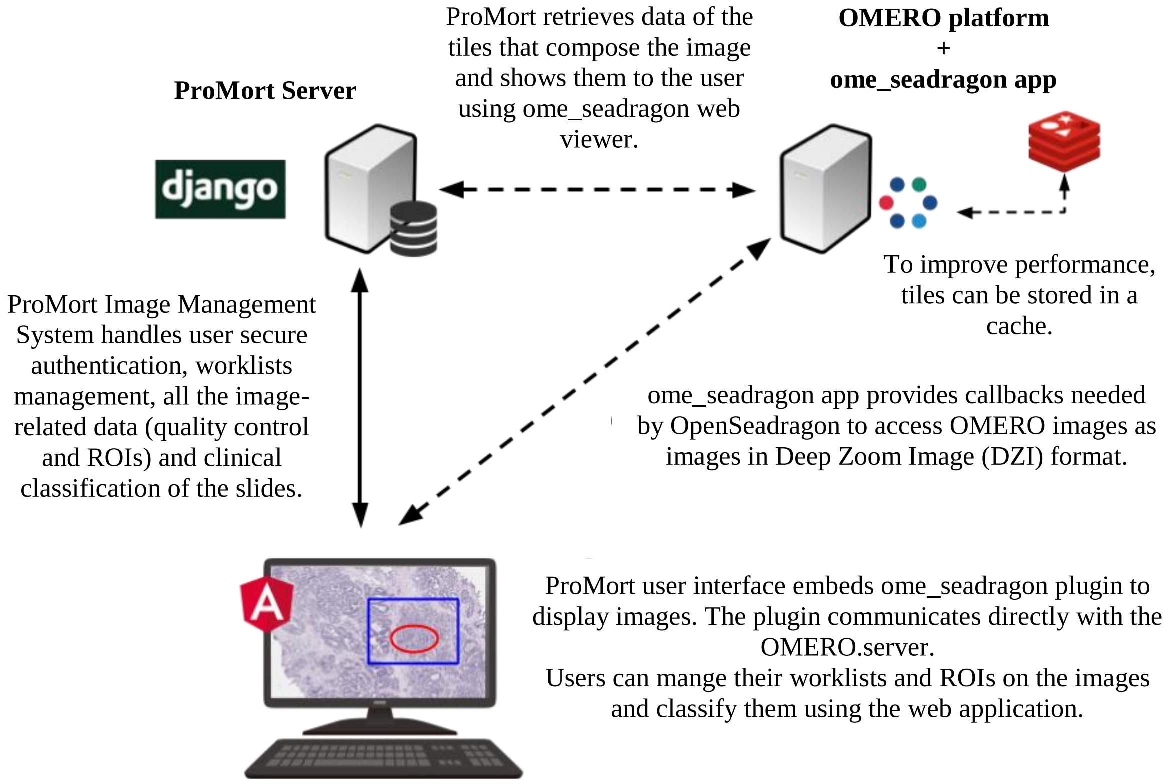

Figure 1. A simplified schematic representation of the virtual microscopy system

Abbreviations: OMERO, The Open Microscopy Environment Remote Objects; ROIs, Regions of interest

Notes: Histology slide image is a cropped version of the Cancer Genome Atlas (TCGA-PRAD) image which is freely available at the Genomic Data Commons (GDC) portal and is a part of the Open Access data tier (the dbGaP accession phs000178.v11.p8); Server icon – "Gorilla-server.svg", a server icon in the Gorilla icon theme style by George Boukeas licensed under CC-BY-SA 3.0. Taken from Wikimedia commons; Django logo – "Django logo" is a trademark of Django Software Foundation, licensed under CC-BY-SA 3.0. Taken from Django community; Angular logo – "Angular logo" by the Angular team, licensed under CC BY 4.0. Taken from Angular presskit; OMERO mark – "OME mark", by the Open Microscopy Environment, licensed under CC BY 4.0. Taken from OME artwork; Redis logo – "Redis logo"- is a trademark of Redis Labs Ltd, licensed under the 3-Clause BSD license. Any rights therein are reserved to Redis Labs Ltd. Any use by CRS4 is for referential purposes only and does not indicate any sponsorship, endorsement or affiliation between Redis and CRS4

References:

1 Allan, C. *et al.* OMERO: flexible, model-driven data management for experimental biology. *Nat Methods* **9**, 245-253, doi:10.1038/nmeth.1896 (2012).

2 Lianas, L. *et al.* CyTest - An Innovative Open-source Platform for Training and Testing in Cythopathology. *Procd Soc Behv* **228**, 674-681, doi:10.1016/j.sbspro.2016.07.103 (2016).

3 Goode, A., Gilbert, B., Harkes, J., Jukic, D. & Satyanarayanan, M. OpenSlide: A vendor-neutral software foundation for digital pathology. *J Pathol Inform* **4**, 27, doi:10.4103/2153-3539.119005 (2013).

Supplementary Table S1. Histopathological characteristics evaluated on the slide level for 352 slides which were evaluated by both reviewers using both the light and virtual microscopy

	Light microscopy			Virtual microscopy		
	Reviewer 1.1	Reviewer 1.2	Reviewer 2	Reviewer 1.1	Reviewer 1.2	Reviewer 2
	N (%)	N (%)	N (%)	N (%)	N (%)	N (%)
Acute inflammation						
No	325 (92.33)	325 (92.33)	335 (95.17)	324 (92.05)	322 (92.00)	335 (95.99)
Yes	27 (7.67)	27 (7.67)	17 (4.83)	28 (7.95)	28 (8.00)	14 (4.01)
Missing	-	-	-	-	2	3
Chronic inflammation						
No	215 (61.08)	218 (61.93)	247 (70.17)	122 (34.66)	132 (37.71)	224 (64.18)
Yes	137 (38.92)	134 (38.07)	105 (29.83)	230 (65.34)	218 (62.29)	125 (35.82)
Missing	-	-	-	-	2	3
High-grade prostatic intraepithelial neoplasia						
No	329 (93.47)	336 (95.45)	335 (95.17)	317 (90.06)	325 (92.86)	333 (95.42)
Yes	23 (6.53)	16 (4.55)	17 (4.83)	35 (9.94)	25 (7.14)	16 (4.58)
Missing	-	-	-	-	2	3
Postatrophic hyperplasia						
No	339 (96.31)	343 (97.44)	336 (95.45)	337 (95.74)	339 (96.86)	339 (97.13)
Yes	13 (3.69)	9 (2.56)	16 (4.55)	15 (4.26)	11 (3.14)	10 (2.87)
Missing	-	-	-	-	2	3
Periglandular inflammation						
No	259 (73.58)	269 (76.42)	307 (87.22)	199 (56.53)	211 (60.29)	273 (78.22)
Yes	93 (26.42)	83 (23.58)	45 (12.78)	153 (43.47)	139 (39.71)	76 (21.78)
Missing	-	-	-	-	2	3
Intraglandular inflammation						
No	322 (91.48)	321 (91.19)	335 (95.17)	305 (86.65)	314 (89.71)	333 (95.42)
Yes	30 (8.52)	31 (8.81)	17 (4.83)	47 (13.35)	36 (10.29)	16 (4.58)
Missing	-	-	-	-	2	3
Stromal inflammation						
No	279 (79.26)	258 (73.30)	257 (73.01)	187 (53.13)	207 (59.14)	258 (73.93)
Yes	73 (20.74)	94 (26.70)	95 (26.99)	165 (46.88)	143 (40.86)	91 (26.07)

Abbreviations: N, Sample size; Reviewer 1.1, First review by Reviewer 1; Reviewer 1.2, Second review by Reviewer 1

Supplementary Table S2. Case-level summary of histopathological characteristics for 60 cases

	Light microscopy			Virtual microscopy		
	Reviewer 1.1	Reviewer 1.2	Reviewer 2	Reviewer 1.1	Reviewer 1.2	Reviewer 2
	N (%)	N (%)	N (%)	N (%)	N (%)	N (%)
Biopsy core length (mm)[1]						
Mean (SD)	73.53 (26.10)	72.33 (25.04)	72.45 (26.36)	77.55 (25.47)	78.09 (26.33)	75.11 (25.30)
Positive core						
No	3 (5.00)	2 (3.33)	3 (5.00)	2 (3.33)	2 (3.33)	2 (3.33)
Yes	57 (95.00)	58 (96.67)	57 (95.00)	58 (96.67)	58 (96.67)	58 (96.67)
Tumor length (mm)[1]						
Mean (SD)	34.49 (26.70)	32.38 (26.32)	34.60 (27.10)	30.35 (26.17)	30.95 (25.97)	29.20 (26.85)
Primary Gleason Grade						
3	16 (28.07)	17 (29.31)	15 (26.32)	16 (27.59)	16 (27.59)	18 (31.03)
4	34 (59.65)	40 (68.97)	32 (56.14)	37 (63.79)	36 (62.07)	37 (63.79)
5	7 (12.28)	1 (1.72)	10 (17.54)	5 (8.62)	6 (10.34)	3 (5.17)
Secondary Gleason grade						
3	12 (24.14)	14 (24.14)	12 (21.05)	15 (25.86)	12 (20.69)	15 (25.86)
4	28 (41.38)	24 (41.38)	29 (50.88)	28 (48.28)	28 (48.28)	25 (43.10)
5	17 (34.48)	20 (34.48)	16 (28.07)	15 (25.86)	18 (31.03)	18 (31.03)
Gleason score[2]						
6	7 (12.28)	9 (15.52)	7 (12.28)	8 (13.79)	8 (13.79)	10 (17.24)
7	14 (24.56)	13 (22.41)	13 (22.81)	15 (25.86)	12 (20.69)	13 (22.41)
8	14 (24.56)	15 (25.86)	14 (24.56)	15 (25.86)	15 (20.69)	14 (24.14)
9	20 (35.09)	21 (36.21)	20 (35.09)	20 (34.48)	22 (37.93)	21 (36.21)
10	2 (3.51)		3 (5.26)		1 (1.72)	
Gleason Grade Groups[3]						
1	7 (12.28)	9 (15.52)	7 (12.28)	8 (13.79)	8 (13.79)	10 (17.24)
2	9 (15.79)	8 (13.79)	8 (14.04)	8 (13.79)	8 (13.79)	18 (13.79)
3	5 (8.77)	5 (8.62)	5 (8.77)	7 (12.07)	4 (6.90)	5 (8.62)
4	14 (24.56)	15 (25.86)	14 (24.56)	15 (25.86)	15 (25.86)	14 (24.14)
5	22 (38.60)	21 (36.21)	23 (40.35)	20 (34.48)	23 (39.66)	21 (36.21)
Perineural invasion[4]						
No	35 (61.40)	40 (68.97)	36 (63.16)	40 (68.97)	39 (67.24)	39 (67.24)
Yes	22 (38.60)	18 (31.03)	21 (36.84)	18 (31.03)	19 (32.76)	19 (32.76)

Abbreviations: N, Sample size; SD, Standard deviation; Reviewer 1.1, First review by Reviewer 1; Reviewer 1.2, Second review by Reviewer 1

[1] Sum of all the core level measurements per case

[2] The highest core-level Gleason score

[3] The highest Gleason core-level Grade Group

[4] Perineural invasion present on at least one core for one case

Supplementary Table S3. Reasons for exclusion of diagnostic slides

	Light microscopy			Virtual microscopy		
	Reviewer 1.1 N=362	Reviewer 1.2 N=362	Reviewer 2 N=362	Reviewer 1.1 N=417	Reviewer 1.2 N=417	Reviewer 2 N=417
	N (%)	N (%)	N (%)	N (%)	N (%)	N (%)
Rejection reason						
Technically inadequate	-	-	-	2 (0.48)	1 (0.24)	2 (0.24)
No prostate tissue	4 (1.10)	6 (1.66)	5 (1.38)	5 (1.20)	5 (1.20)	6 (1.20)
No tissue	3 (0.83)	-	2 (0.55)	1 (0.24)	1 (0.24)	1 (0.24)
Duplicate slide	-	-	-	54 (12.95)	55 (12.95)	54 (12.47)

Abbreviations: N, Sample size; Reviewer 1.1, First review by Reviewer 1; Reviewer 1.2, Second review by Reviewer 1

Supplementary Figure S1. Bland and Altman plot of the intra-method intra-observer agreement for biopsy core length measured in millimeters, evaluated on the core level

Abbreviations: Light 1.1, First review by Reviewer 1 on light microscopy; Light 1.2, Second review by Reviewer 1 on light microscopy; Virtual 1.1, First review by Reviewer 1 on virtual microscopy; Virtual 1.2, Second review by Reviewer 1 on virtual microscopy

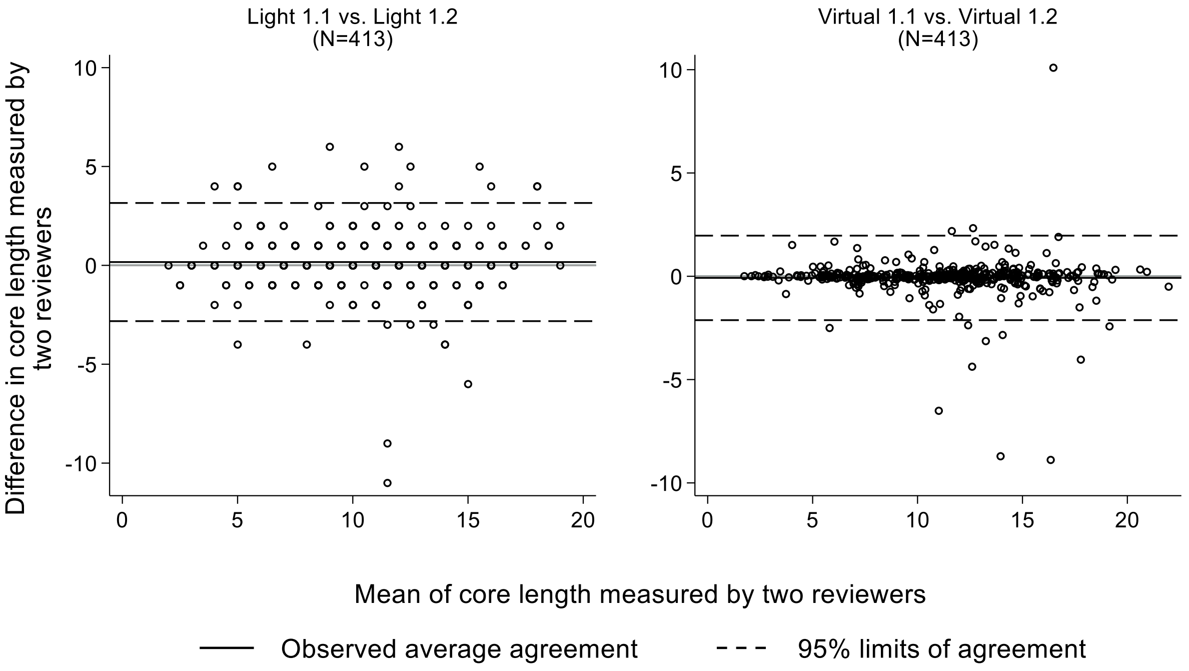

Supplementary Figure S2. Bland and Altman plot of the intra-method intra-observer agreement for length of tumor in the biopsy core measured in millimeters, evaluated on the core level

Abbreviations: Light 1.1, First review by Reviewer 1 on light microscopy; Light 1.2, Second review by Reviewer 1 on light microscopy; Virtual 1.1, First review by Reviewer 1 on virtual microscopy; Virtual 1.2, Second review by Reviewer 1 on virtual microscopy

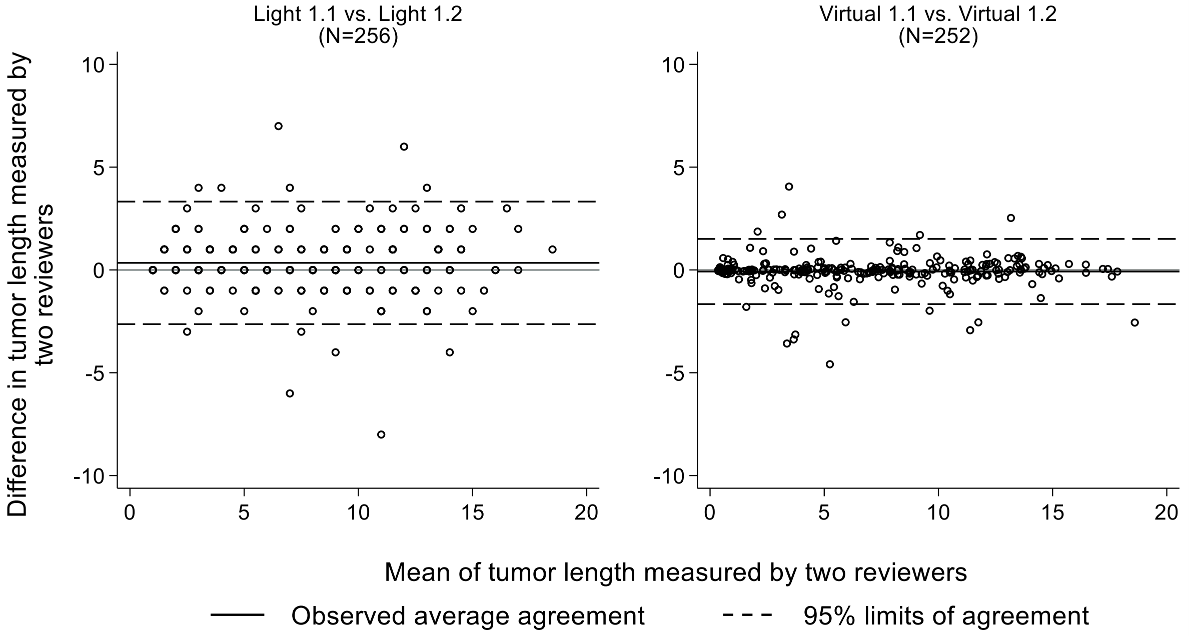

Supplementary Figure S3. Intra-method intra-observer agreement for characteristics evaluated on the core level

Abbreviations: Light 1.1, First review by Reviewer 1 on light microscopy; Light 1.2, Second review by Reviewer 1 on light microscopy; Virtual 1.1, First review by Reviewer 1 on virtual microscopy; Virtual 1.2, Second review by Reviewer 1 on virtual microscopy

Comparison	Sample size	Cohen's kappa (95% CI)
Primary Gleason pattern		
Light 1.1 vs. Light 1.2	256	0.79 (0.72, 0.86)
Virtual 1.1 vs. Virtual 1.2	253	0.84 (0.78, 0.91)
Secondary Gleason pattern		
Light 1.1 vs. Light 1.2	256	0.67 (0.60, 0.75)
Virtual 1.1 vs. Virtual 1.2	253	0.66 (0.58, 0.73)
Gleason score		
Light 1.1 vs. Light 1.2	256	0.82 (0.78, 0.87)
Virtual 1.1 vs. Virtual 1.2	253	0.81 (0.77, 0.86)
Gleason Grade Groups		
Light 1.1 vs. Light 1.2	256	0.85 (0.81, 0.88)
Virtual 1.1 vs. Virtual 1.2	253	0.84 (0.80, 0.88)
Cribriform pattern		
Light 1.1 vs. Light 1.2	256	0.68 (0.59, 0.77)
Virtual 1.1 vs. Virtual 1.2	253	0.79 (0.72, 0.87)
Poorly formed glands		
Light 1.1 vs. Light 1.2	256	0.54 (0.43, 0.65)
Virtual 1.1 vs. Virtual 1.2	253	0.67 (0.57, 0.77)
Comedonecrosis		
Light 1.1 vs. Light 1.2	256	0.81 (0.63, 0.99)
Virtual 1.1 vs. Virtual 1.2	253	0.80 (0.64, 0.97)
Perineural invasion		
Light 1.1 vs. Light 1.2	256	0.61 (0.49, 0.73)
Virtual 1.1 vs. Virtual 1.2	253	0.60 (0.47, 0.73)
Intraductal carcinoma		
Light 1.1 vs. Light 1.2	256	0.28 (-0.16, 0.72)
Virtual 1.1 vs. Virtual 1.2	253	0.53 (0.17, 0.90)
Mucinous carcinoma		
Light 1.1 vs. Light 1.2	256	0.66 (0.30, 1.00)
Virtual 1.1 vs. Virtual 1.2	253	0.89 (0.67, 1.00)

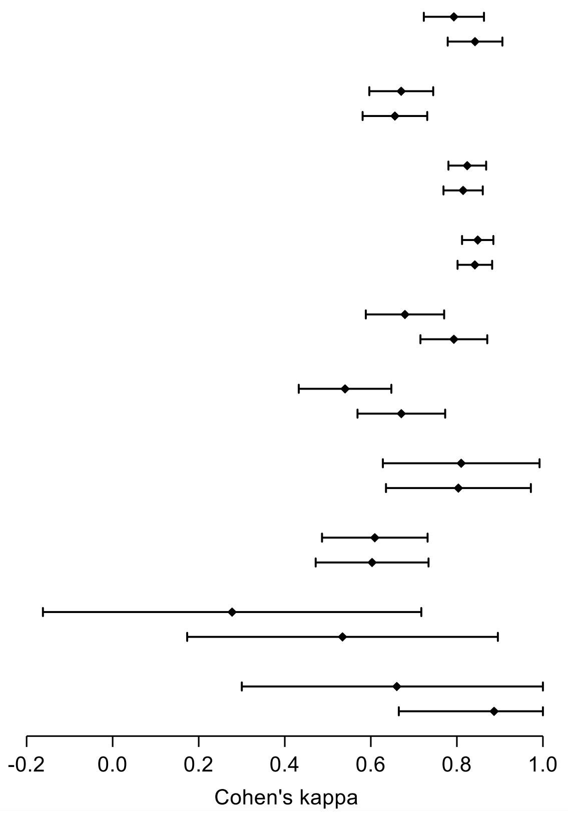

Supplementary Figure S4. Intra-method intra-observer agreement for characteristics evaluated on the slide level

Abbreviations: Light 1.1, First review by Reviewer 1 on light microscopy; Light 1.2, Second review by Reviewer 1 on light microscopy; Virtual 1.1, First review by Reviewer 1 on virtual microscopy; Virtual 1.2, Second review by Reviewer 1 on virtual microscopy

Comparison	Sample size	Cohen's kappa (95% CI)
Acute inflammation		
Light 1.1 vs. Light 1.2	352	0.52 (0.35, 0.69)
Virtual 1.1 vs. Virtual 1.2	350	0.69 (0.55, 0.83)
Chronic inflammation		
Light 1.1 vs. Light 1.2	352	0.47 (0.37, 0.56)
Virtual 1.1 vs. Virtual 1.2	350	0.66 (0.58, 0.74)
High-grade prostatic intraepithelial neoplasia		
Light 1.1 vs. Light 1.2	352	0.43 (0.23, 0.63)
Virtual 1.1 vs. Virtual 1.2	350	0.53 (0.37, 0.69)
Intraglandular inflamation		
Light 1.1 vs. Light 1.2	352	0.52 (0.36, 0.68)
Virtual 1.1 vs. Virtual 1.2	350	0.69 (0.57, 0.81)
Postatrophic hyperplasia		
Light 1.1 vs. Light 1.2	352	0.53 (0.28, 0.79)
Virtual 1.1 vs. Virtual 1.2	350	0.60 (0.37, 0.83)
Periglandular inflamation		
Light 1.1 vs. Light 1.2	352	0.50 (0.40, 0.60)
Virtual 1.1 vs. Virtual 1.2	350	0.62 (0.54, 0.71)
Stromal inflammation		
Light 1.1 vs. Light 1.2	352	0.32 (0.21, 0.43)
Virtual 1.1 vs. Virtual 1.2	350	0.59 (0.50, 0.67)

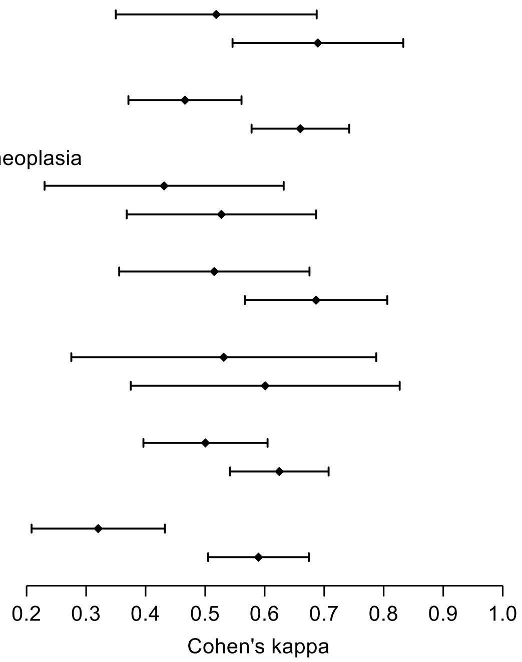

Supplementary Figure S5. Bland and Altman plot of the intra-method intra-observer agreement for the percentage of Gleason pattern 4, evaluated on the core level

Abbreviations: Light 1.1, First review by Reviewer 1 on light microscopy; Light 1.2, Second review by Reviewer 1 on light microscopy; Virtual 1.1, First review by Reviewer 1 on virtual microscopy; Virtual 1.2, Second review by Reviewer 1 on virtual microscopy

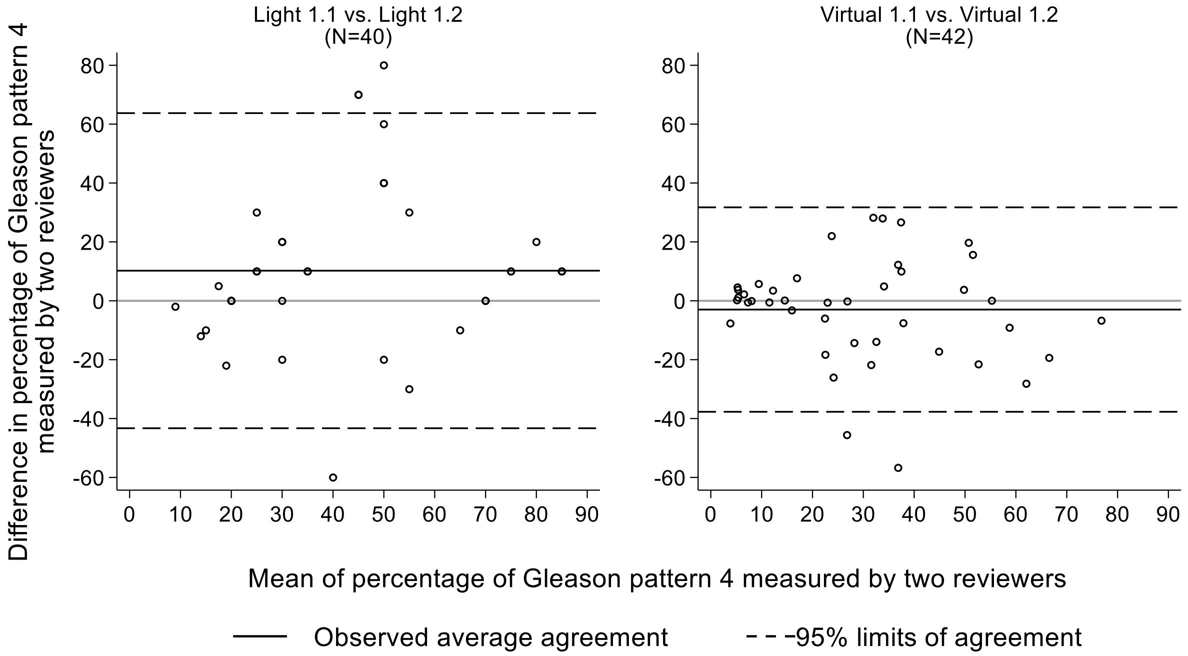

Supplementary Figure S6. Bland and Altman plot of the intra-method inter-observer agreement for biopsy core length measured in millimeters, evaluated on the core level

Abbreviations: Light1.1, First review by Reviewer 1 on light microscopy; Light 2, Reviewer 2 on light microscopy; Light 1.2, Second review by Reviewer 1 on light microscopy; Virtual 1.1, First review by Reviewer 1 on virtual microscopy; Virtual 2, Reviewer 2 on virtual microscopy; Virtual 1.2, Second review by Reviewer 1 on virtual microscopy

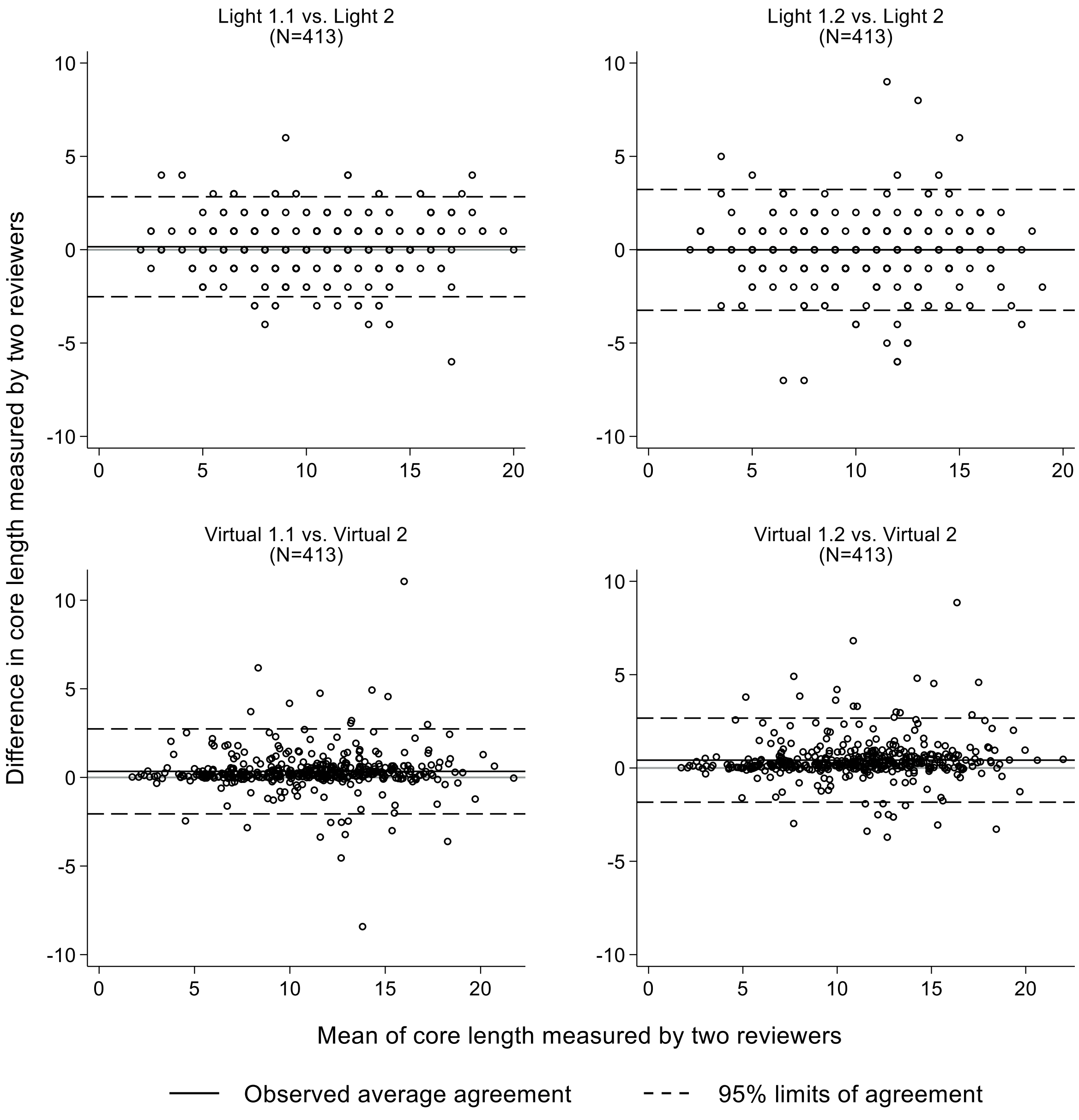

Supplementary Figure S7. Bland and Altman plot of the intra-method inter-observer agreement for the length of tumor in the biopsy core measured in millimeters, evaluated on the core level

Abbreviations: Light 1.1, First review by Reviewer 1 on light microscopy; Light 2, Reviewer 2 on light microscopy; Light 1.2, Second review by Reviewer 1 on light microscopy; Virtual 1.1, First review by Reviewer 1 on virtual microscopy; Virtual 2, Reviewer 2 on virtual microscopy; Virtual 1.2, Second review by Reviewer 1 on virtual microscopy

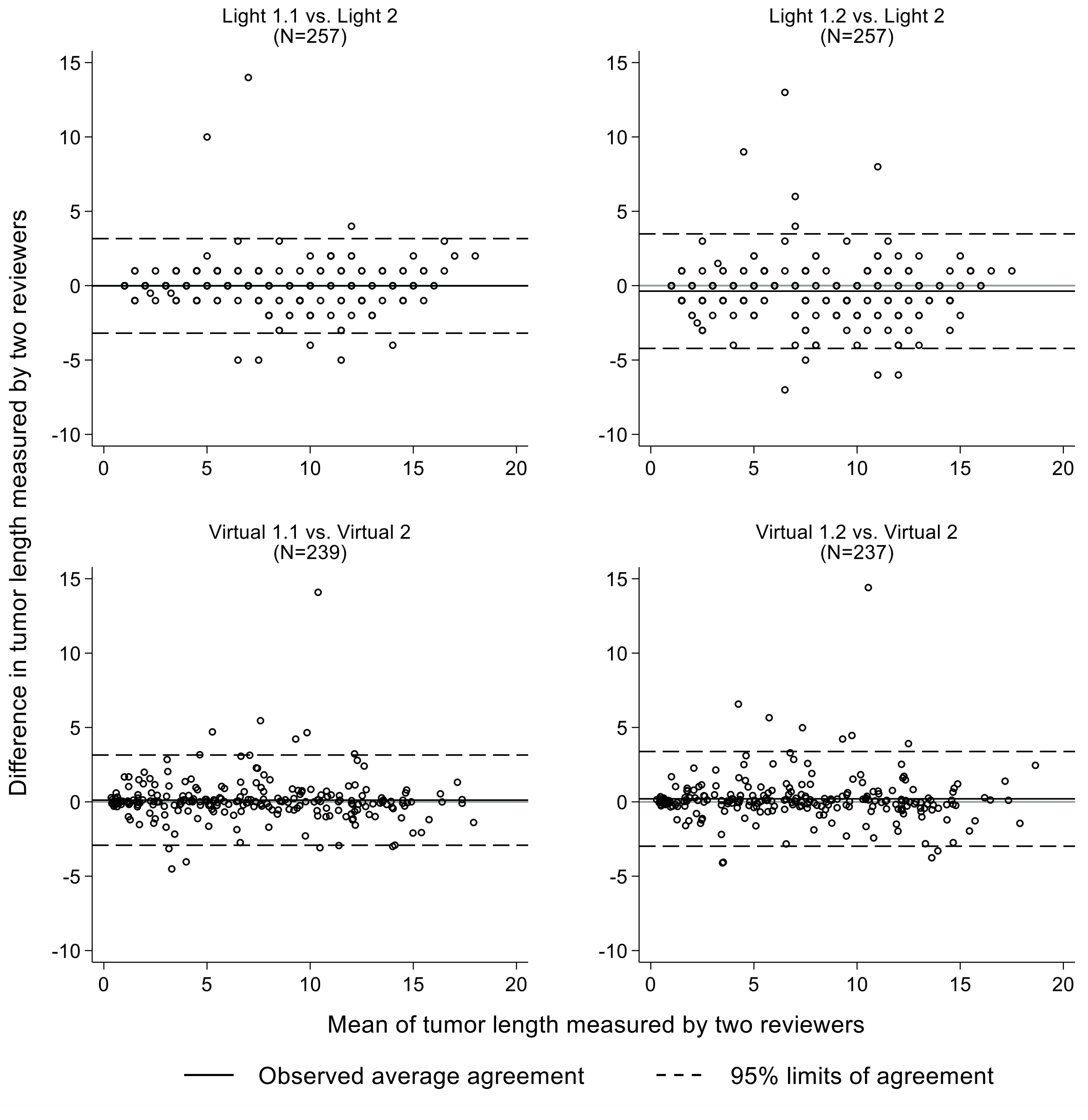

Supplementary Figure S8. Intra-method inter-observer agreement for characteristics evaluated on the core level

Abbreviations: Light 1.1, First review by Reviewer 1 on light microscopy; Light 2, Reviewer 2 on light microscopy; Light 1.2, Second review by Reviewer 1 on light microscopy; Virtual 1.1, First review by Reviewer 1 on virtual microscopy; Virtual 2, Reviewer 2 on virtual microscopy; Virtual 1.2, Second review by Reviewer 1 on virtual microscopy;

Comparison	Sample size	Cohen's kappa (95% CI)
Primary Gleason pattern		
Light 1.1 vs. Light 2	257	0.89 (0.84, 0.94)
Light 1.2 vs. Light 2	255	0.72 (0.65, 0.79)
Virtual 1.1 vs. Virtual 2	243	0.80 (0.73, 0.87)
Virtual 1.2 vs. Virtual 2	242	0.78 (0.70, 0.85)
Secondary Gleason pattern		
Light 1.1 vs. Light 2	257	0.74 (0.68, 0.81)
Light 1.2 vs. Light 2	255	0.58 (0.50, 0.66)
Virtual 1.1 vs. Virtual 2	243	0.68 (0.61, 0.75)
Virtual 1.2 vs. Virtual 2	242	0.67 (0.60, 0.74)
Gleason score		
Light 1.1 vs. Light 2	257	0.86 (0.83, 0.90)
Light 1.2 vs. Light 2	255	0.76 (0.71, 0.81)
Virtual 1.1 vs. Virtual 2	243	0.81 (0.77, 0.86)
Virtual 1.2 vs. Virtual 2	242	0.81 (0.76, 0.86)
Gleason Grade Groups		
Light 1.1 vs. Light 2	257	0.89 (0.86, 0.92)
Light 1.2 vs. Light 2	255	0.80 (0.76, 0.84)
Virtual 1.1 vs. Virtual 2	243	0.83 (0.80, 0.87)
Virtual 1.2 vs. Virtual 2	242	0.83 (0.78, 0.87)
Cribriform pattern		
Light 1.1 vs. Light 2	257	0.58 (0.48, 0.68)
Light 1.2 vs. Light 2	255	0.48 (0.38, 0.59)
Virtual 1.1 vs. Virtual 2	243	0.61 (0.51, 0.71)
Virtual 1.2 vs. Virtual 2	242	0.61 (0.51, 0.72)
Poorly formed glands		
Light 1.1 vs. Light 2	257	0.61 (0.52, 0.71)
Light 1.2 vs. Light 2	255	0.56 (0.45, 0.66)
Virtual 1.1 vs. Virtual 2	243	0.61 (0.50, 0.71)
Virtual 1.2 vs. Virtual 2	242	0.54 (0.43, 0.65)
Comedonecrosis		
Light 1.1 vs. Light 2	257	0.64 (0.46, 0.83)
Light 1.2 vs. Light 2	255	0.50 (0.29, 0.71)
Virtual 1.1 vs. Virtual 2	243	0.51 (0.27, 0.75)
Virtual 1.2 vs. Virtual 2	242	0.57 (0.34, 0.80)
Perineural invasion		
Light 1.1 vs. Light 2	257	0.90 (0.84, 0.96)
Light 1.2 vs. Light 2	255	0.61 (0.49, 0.73)
Virtual 1.1 vs. Virtual 2	243	0.38 (0.22, 0.53)
Virtual 1.2 vs. Virtual 2	242	0.45 (0.30, 0.60)
Intraductal carcinoma		
Light 1.1 vs. Light 2	257	0.24 (-0.03, 0.51)
Light 1.2 vs. Light 2	255	0.19 (-0.06, 0.43)
Virtual 1.1 vs. Virtual 2	243	0.14 (-0.15, 0.43)
Virtual 1.2 vs. Virtual 2	242	0.43 (0.02, 0.84)
Mucinous carcinoma		
Light 1.1 vs. Light 2	257	0.59 (0.33, 0.84)
Light 1.2 vs. Light 2	255	0.34 (0.05, 0.63)
Virtual 1.1 vs. Virtual 2	243	0.27 (-0.17, 0.72)
Virtual 1.2 vs. Virtual 2	242	0.23 (-0.17, 0.64)

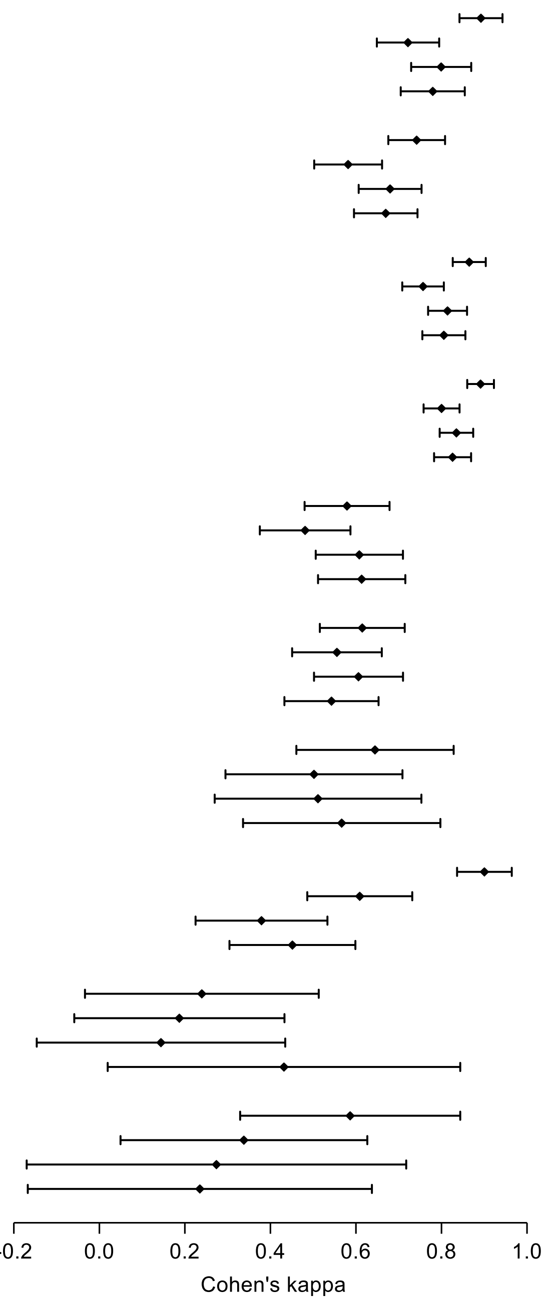

Supplementary Figure S9. Intra-method inter-observer agreement for characteristics evaluated on the slide level

Abbreviations: Light 1.1, First review by Reviewer 1 on light microscopy; Light 2, Reviewer 2 on light microscopy; Light 1.2, Second review by Reviewer 1 on light microscopy; Virtual 1.1, First review by Reviewer 1 on virtual microscopy; Virtual 2, Reviewer 2 on virtual microscopy; Virtual 1.2, Second review by Reviewer 1 on virtual microscopy;

Comparison	Sample size	Cohen's kappa (95% CI)
Acute inflammation		
Light 1.1 vs. Light 2	352	0.71 (0.56, 0.87)
Light 1.2 vs. Light 2	352	0.42 (0.23, 0.61)
Virtual 1.1 vs. Virtual 2	349	0.45 (0.26, 0.64)
Virtual 1.2 vs. Virtual 2	347	0.40 (0.20, 0.59)
Chronic inflammation		
Light 1.1 vs. Light 2	352	0.68 (0.60, 0.76)
Light 1.2 vs. Light 2	352	0.49 (0.40, 0.59)
Virtual 1.1 vs. Virtual 2	349	0.36 (0.29, 0.44)
Virtual 1.2 vs. Virtual 2	347	0.31 (0.22, 0.39)
High-grade prostatic intraepithelial neoplasia		
Light 1.1 vs. Light 2	352	0.68 (0.51, 0.85)
Light 1.2 vs. Light 2	352	0.27 (0.06, 0.48)
Virtual 1.1 vs. Virtual 2	349	0.23 (0.06, 0.39)
Virtual 1.2 vs. Virtual 2	347	0.15 (-0.02, 0.32)
Intraglandular inflamation		
Light 1.1 vs. Light 2	352	0.61 (0.45, 0.78)
Light 1.2 vs. Light 2	352	0.38 (0.20, 0.56)
Virtual 1.1 vs. Virtual 2	349	0.34 (0.18, 0.49)
Virtual 1.2 vs. Virtual 2	347	0.38 (0.21, 0.56)
Postatrophic hyperplasia		
Light 1.1 vs. Light 2	352	0.60 (0.39, 0.82)
Light 1.2 vs. Light 2	352	0.46 (0.22, 0.71)
Virtual 1.1 vs. Virtual 2	349	0.54 (0.31, 0.78)
Virtual 1.2 vs. Virtual 2	347	0.66 (0.42, 0.89)
Periglandular inflamation		
Light 1.1 vs. Light 2	352	0.40 (0.29, 0.51)
Light 1.2 vs. Light 2	352	0.29 (0.17, 0.41)
Virtual 1.1 vs. Virtual 2	349	0.39 (0.30, 0.48)
Virtual 1.2 vs. Virtual 2	347	0.34 (0.25, 0.44)
Stromal inflammation		
Light 1.1 vs. Light 2	352	0.49 (0.38, 0.59)
Light 1.2 vs. Light 2	352	0.43 (0.32, 0.53)
Virtual 1.1 vs. Virtual 2	349	0.28 (0.19, 0.37)
Virtual 1.2 vs. Virtual 2	347	0.30 (0.20, 0.39)

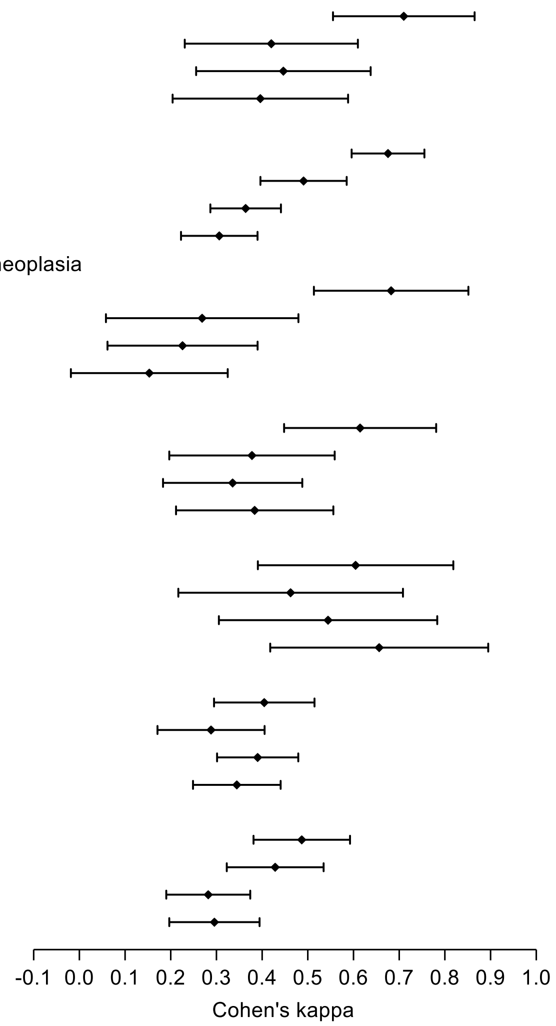

Supplementary Figure S10. Bland and Altman plot of the intra-method inter-observer agreement for the percentage of Gleason pattern 4, evaluated on the core level

Abbreviations: Light 1.1, First review by Reviewer 1 on light microscopy; Light 2, Reviewer 2 on light microscopy; Light 1.2, Second review by Reviewer 1 on light microscopy; Virtual 1.1, First review by Reviewer 1 on virtual microscopy; Virtual 2, Reviewer 2 on virtual microscopy; Virtual 1.2, Second review by Reviewer 1 on virtual microscopy

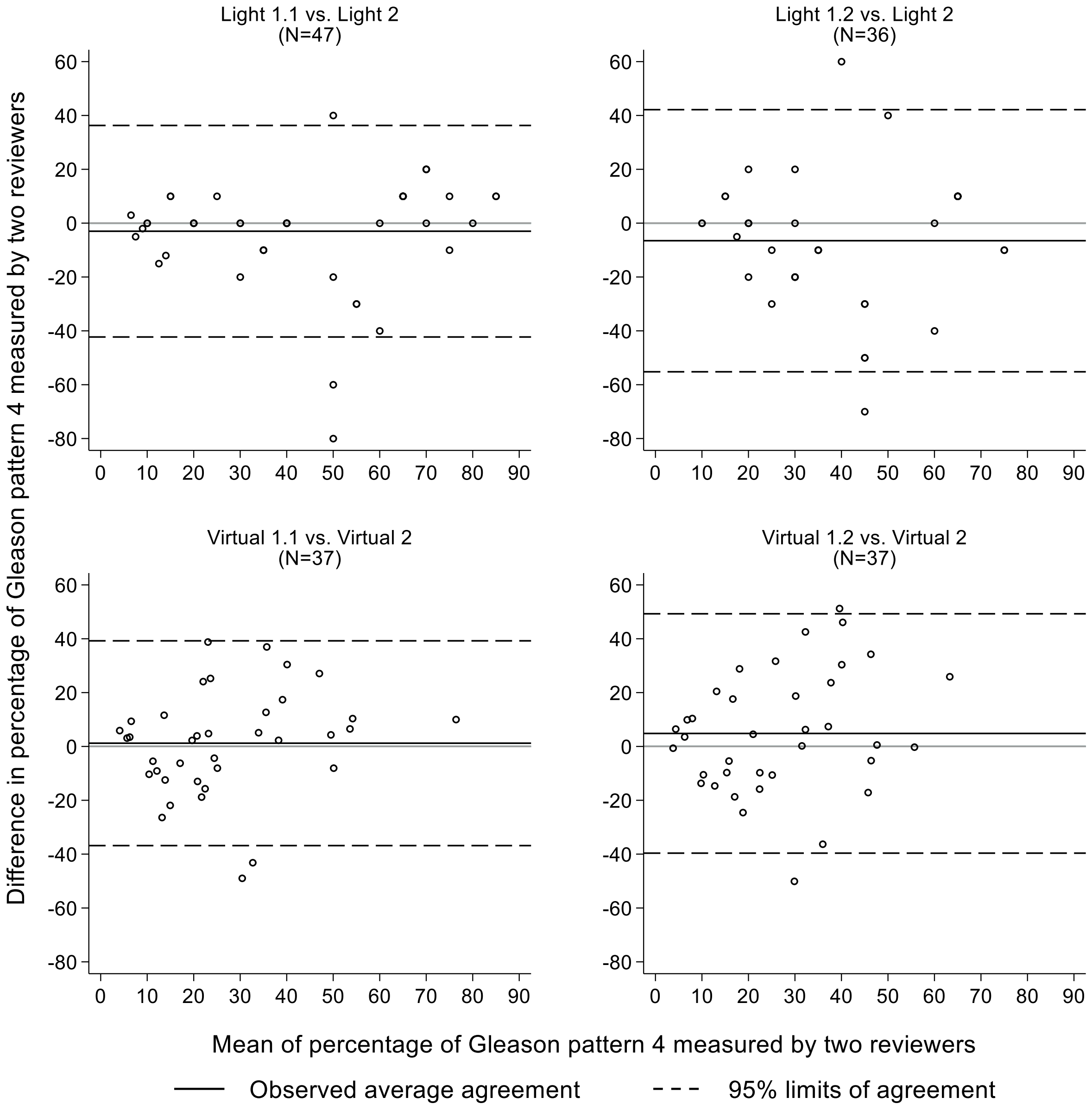

Supplementary Figure S11. Bland and Altman plot of the inter-method intra-observer agreement for the biopsy core length measured in millimeters, evaluated on the core level

Abbreviations: Light 1.1, First review by Reviewer 1 on light microscopy; Virtual 1.1, First review by Reviewer 1 on virtual microscopy; Virtual 1.2, Second review by Reviewer 1 on virtual microscopy; Light 2, Reviewer 2 on light microscopy; Virtual 2, Reviewer 2 on virtual microscopy; Light 1.2, Second review by Reviewer 1 on light microscopy

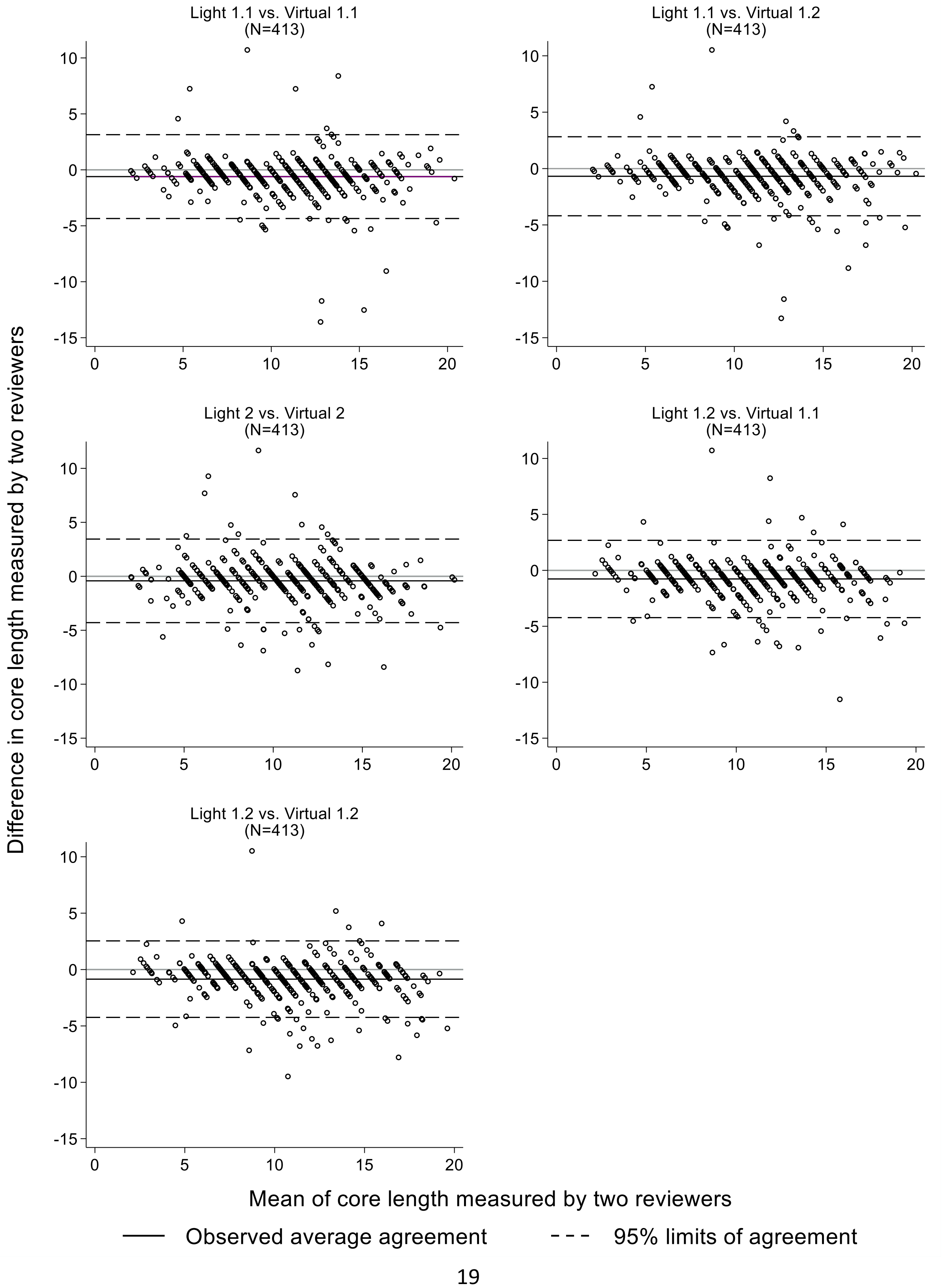

Supplementary Figure S12. Bland and Altman plot of the inter-method intra-observer agreement for the length of tumor in the biopsy core measured in millimeters, evaluated on the core level

Abbreviations: Light 1.1, First review by Reviewer 1 on light microscopy; Virtual 1.1, First review by Reviewer 1 on virtual microscopy; Virtual 1.2, Second review by Reviewer 1 on virtual microscopy; Light 2, Reviewer 2 on light microscopy; Virtual 2, Reviewer 2 on virtual microscopy; Light 1.2, Second review by Reviewer 1 on light microscopy

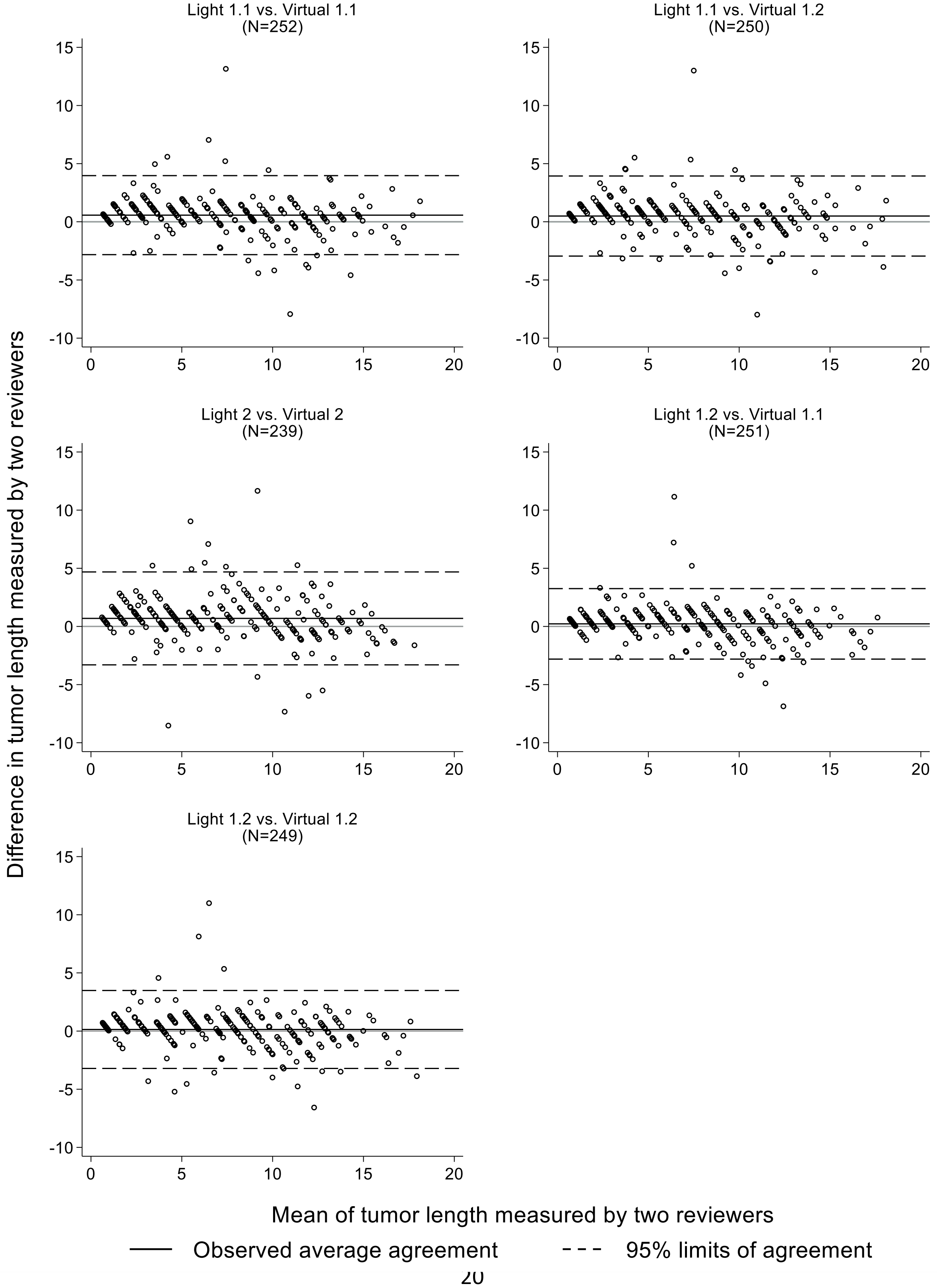

Supplementary Figure S13. Inter-method intra-observer agreement for characteristics evaluated on the core level

Abbreviations: Light 1.1, First review by Reviewer 1 on light microscopy; Virtual 1.1, First review by Reviewer 1 on virtual microscopy; Virtual 1.2, Second review by Reviewer 1 on virtual microscopy; Light 2, Reviewer 2 on light microscopy; Virtual 2, Reviewer 2 on virtual microscopy; Light 1.2, Second review by Reviewer 1 on light microscopy

Comparison	Sample size	Cohen's kappa (95% CI)
**Primary Gleason pattern**		
Light 1.1 vs. Virtual 1.1	252	0.88 (0.83, 0.94)
Light 1.1 vs. Virtual 1.2	251	0.86 (0.80, 0.92)
Light 2 vs. Virtual 2	243	0.69 (0.62, 0.77)
Light 1.2 vs. Virtual 1.1	251	0.80 (0.73, 0.87)
Light 1.2 vs. Virtual 1.2	250	0.81 (0.74, 0.88)
**Secondary Gleason pattern**		
Light 1.1 vs. Virtual 1.1	252	0.68 (0.61, 0.76)
Light 1.1 vs. Virtual 1.2	251	0.75 (0.69, 0.82)
Light 2 vs. Virtual 2	243	0.59 (0.51, 0.67)
Light 1.2 vs. Virtual 1.1	251	0.68 (0.61, 0.76)
Light 1.2 vs. Virtual 1.2	250	0.69 (0.62, 0.76)
**Gleason score**		
Light 1.1 vs. Virtual 1.1	252	0.84 (0.80, 0.89)
Light 1.1 vs. Virtual 1.2	251	0.85 (0.80, 0.89)
Light 2 vs. Virtual 2	243	0.77 (0.72, 0.82)
Light 1.2 vs. Virtual 1.1	251	0.82 (0.77, 0.87)
Light 1.2 vs. Virtual 1.2	250	0.81 (0.76, 0.86)
**Gleason Grade Groups**		
Light 1.1 vs. Virtual 1.1	252	0.87 (0.84, 0.91)
Light 1.1 vs. Virtual 1.2	251	0.86 (0.82, 0.90)
Light 2 vs. Virtual 2	243	0.81 (0.76, 0.85)
Light 1.2 vs. Virtual 1.1	251	0.83 (0.79, 0.88)
Light 1.2 vs. Virtual 1.2	250	0.83 (0.79, 0.88)
**Cribriform pattern**		
Light 1.1 vs. Virtual 1.1	252	0.64 (0.54, 0.74)
Light 1.1 vs. Virtual 1.2	251	0.57 (0.47, 0.68)
Light 2 vs. Virtual 2	243	0.49 (0.39, 0.60)
Light 1.2 vs. Virtual 1.1	251	0.68 (0.59, 0.78)
Light 1.2 vs. Virtual 1.2	250	0.61 (0.51, 0.71)
**Poorly formed glands**		
Light 1.1 vs. Virtual 1.1	252	0.55 (0.44, 0.66)
Light 1.1 vs. Virtual 1.2	251	0.62 (0.51, 0.72)
Light 2 vs. Virtual 2	243	0.53 (0.42, 0.64)
Light 1.2 vs. Virtual 1.1	251	0.68 (0.58, 0.78)
Light 1.2 vs. Virtual 1.2	250	0.61 (0.51, 0.72)
**Comedonecrosis**		
Light 1.1 vs. Virtual 1.1	252	0.79 (0.61, 0.97)
Light 1.1 vs. Virtual 1.2	251	0.84 (0.68, 0.99)
Light 2 vs. Virtual 2	243	0.45 (0.24, 0.65)
Light 1.2 vs. Virtual 1.1	251	0.68 (0.46, 0.90)
Light 1.2 vs. Virtual 1.2	250	0.74 (0.54, 0.94)
**Perineural invasion**		
Light 1.1 vs. Virtual 1.1	252	0.65 (0.53, 0.77)
Light 1.1 vs. Virtual 1.2	251	0.65 (0.53, 0.77)
Light 2 vs. Virtual 2	243	0.49 (0.36, 0.63)
Light 1.2 vs. Virtual 1.1	251	0.60 (0.47, 0.74)
Light 1.2 vs. Virtual 1.2	250	0.52 (0.38, 0.66)
**Intraductal carcinoma**		
Light 1.1 vs. Virtual 1.1	252	0.44 (0.03, 0.84)
Light 1.1 vs. Virtual 1.2	251	0.33 (-0.16, 0.82)
Light 2 vs. Virtual 2	243	0.07 (-0.12, 0.27)
Light 1.2 vs. Virtual 1.1	251	0.32 (-0.03, 0.67)
Light 1.2 vs. Virtual 1.2	250	0.66 (0.30, 1.00)
**Mucinous carcinoma**		
Light 1.1 vs. Virtual 1.1	252	0.69 (0.37, 1.00)
Light 1.1 vs. Virtual 1.2	251	0.72 (0.42, 1.00)
Light 2 vs. Virtual 2	243	0.10 (-0.12, 0.32)
Light 1.2 vs. Virtual 1.1	251	0.56 (0.12, 1.00)
Light 1.2 vs. Virtual 1.2	250	0.75 (0.40, 1.00)

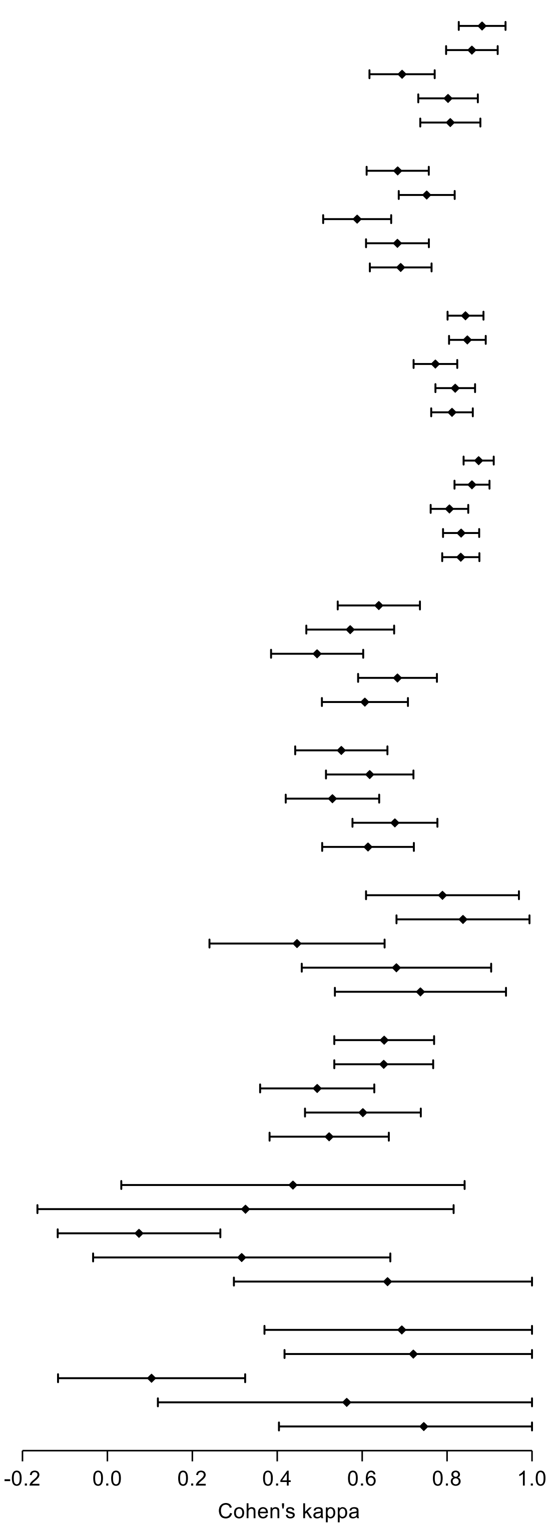

Supplementary Figure S14. Inter-method intra-observer agreement for characteristics evaluated on the slide level

Abbreviations: Light 1.1, First review by Reviewer 1 on light microscopy; Virtual 1.1, First review by Reviewer 1 on virtual microscopy; Virtual 1.2, Second review by Reviewer 1 on virtual microscopy; Light 2, Reviewer 2 on light microscopy; Virtual 2, Reviewer 2 on virtual microscopy; Light 1.2, Second review by Reviewer 1 on light microscopy

Comparison	Sample size	Cohen's kappa (95% CI)
Acute inflammation		
Light 1.1 vs. Virtual 1.1	353	0.51 (0.34, 0.68)
Light 1.1 vs. Virtual 1.2	351	0.63 (0.47, 0.78)
Light 2 vs. Virtual 2	350	0.49 (0.27, 0.72)
Light 1.2 vs. Virtual 1.1	354	0.47 (0.30, 0.64)
Light 1.2 vs. Virtual 1.2	352	0.63 (0.47, 0.78)
Chronic inflammation		
Light 1.1 vs. Virtual 1.1	353	0.38 (0.30, 0.46)
Light 1.1 vs. Virtual 1.2	351	0.40 (0.32, 0.48)
Light 2 vs. Virtual 2	350	0.39 (0.29, 0.49)
Light 1.2 vs. Virtual 1.1	354	0.41 (0.33, 0.49)
Light 1.2 vs. Virtual 1.2	352	0.38 (0.29, 0.46)
High-grade prostatic intraepithelial neoplasia		
Light 1.1 vs. Virtual 1.1	353	0.29 (0.12, 0.45)
Light 1.1 vs. Virtual 1.2	351	0.46 (0.28, 0.65)
Light 2 vs. Virtual 2	350	0.14 (-0.04, 0.32)
Light 1.2 vs. Virtual 1.1	354	0.35 (0.18, 0.53)
Light 1.2 vs. Virtual 1.2	352	0.46 (0.26, 0.65)
Intraglandular inflamation		
Light 1.1 vs. Virtual 1.1	353	0.55 (0.41, 0.69)
Light 1.1 vs. Virtual 1.2	351	0.57 (0.42, 0.72)
Light 2 vs. Virtual 2	350	0.65 (0.46, 0.84)
Light 1.2 vs. Virtual 1.1	354	0.57 (0.43, 0.71)
Light 1.2 vs. Virtual 1.2	352	0.62 (0.48, 0.76)
Postatrophic hyperplasia		
Light 1.1 vs. Virtual 1.1	353	0.33 (0.10, 0.56)
Light 1.1 vs. Virtual 1.2	351	0.48 (0.23, 0.73)
Light 2 vs. Virtual 2	350	0.20 (-0.02, 0.42)
Light 1.2 vs. Virtual 1.1	354	0.40 (0.14, 0.65)
Light 1.2 vs. Virtual 1.2	352	0.59 (0.33, 0.85)
Periglandular inflamation		
Light 1.1 vs. Virtual 1.1	353	0.46 (0.37, 0.55)
Light 1.1 vs. Virtual 1.2	351	0.34 (0.24, 0.44)
Light 2 vs. Virtual 2	350	0.32 (0.20, 0.44)
Light 1.2 vs. Virtual 1.1	354	0.44 (0.35, 0.53)
Light 1.2 vs. Virtual 1.2	352	0.44 (0.34, 0.53)
Stromal inflammation		
Light 1.1 vs. Virtual 1.1	353	0.29 (0.21, 0.38)
Light 1.1 vs. Virtual 1.2	351	0.34 (0.24, 0.43)
Light 2 vs. Virtual 2	350	0.36 (0.25, 0.47)
Light 1.2 vs. Virtual 1.1	354	0.41 (0.32, 0.50)
Light 1.2 vs. Virtual 1.2	352	0.36 (0.26, 0.46)

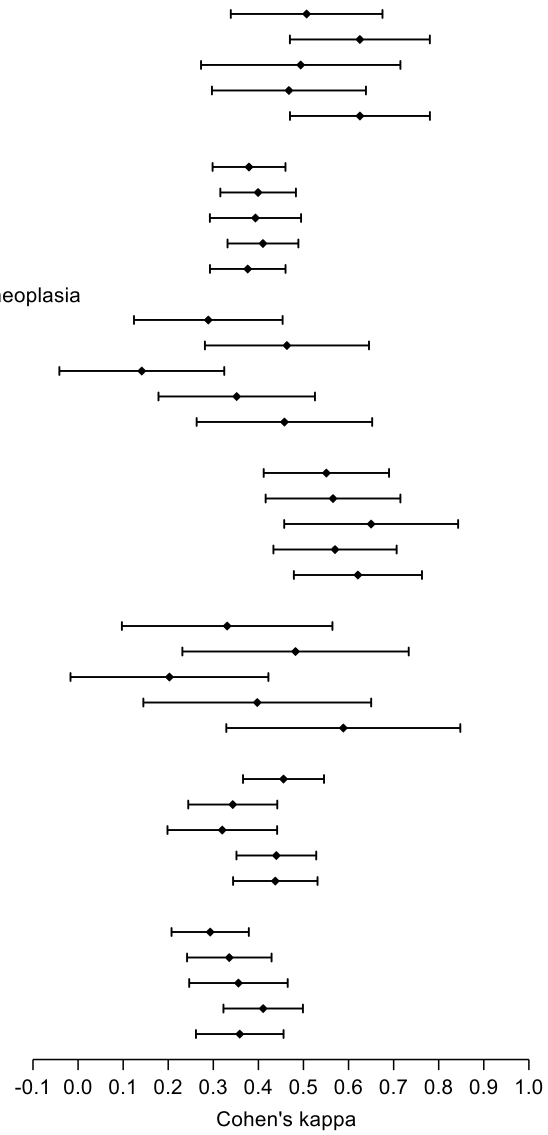

Supplementary Figure S15. Bland and Altman plot of the inter-method intra-observer agreement for the percentage of Gleason pattern 4, evaluated on the core level

Abbreviations: Light 1.1, First review by Reviewer 1 on light microscopy; Virtual 1.1, First review by Reviewer 1 on virtual microscopy; Virtual 1.2, Second review by Reviewer 1 on virtual microscopy; Light 2, Reviewer 2 on light microscopy; Virtual 2, Reviewer 2 on virtual microscopy; Light 1.2, Second review by Reviewer 1 on light microscopy

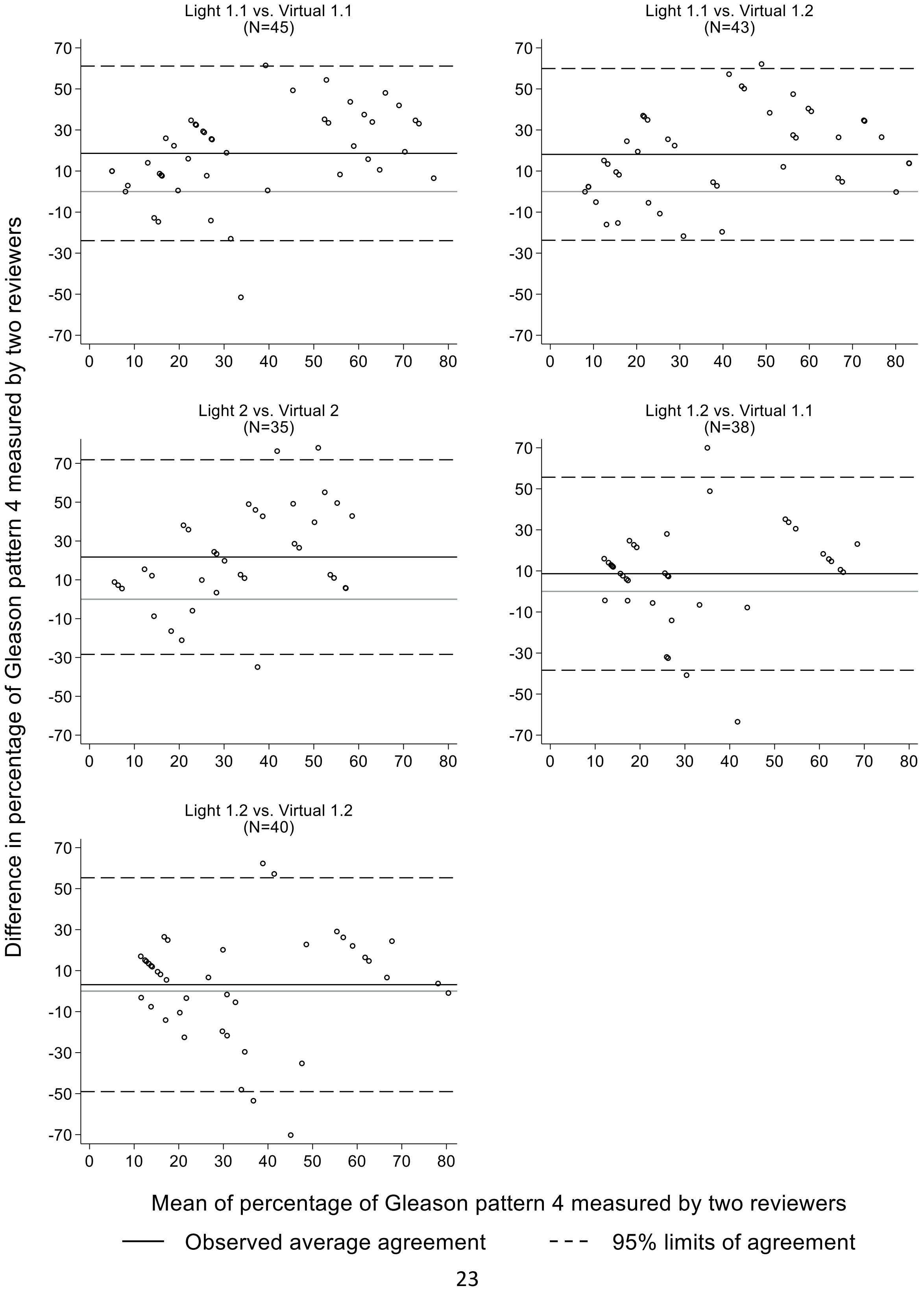

Supplementary Figure S16. Bland and Altman plot of the inter-method inter-observer agreement for the biopsy core length measured in millimeters, evaluated on the core level

Abbreviations: Light 1.1, First review by Reviewer 1 on light microscopy; Virtual 2, Reviewer 2 on virtual microscopy; Light 2, Reviewer 2 on light microscopy; Virtual 1.1, First review by Reviewer 1 on virtual microscopy; Virtual 1.2, Second review by Reviewer 1 on virtual microscopy; Light 1.2, Second review by Reviewer 1 on light microscopy

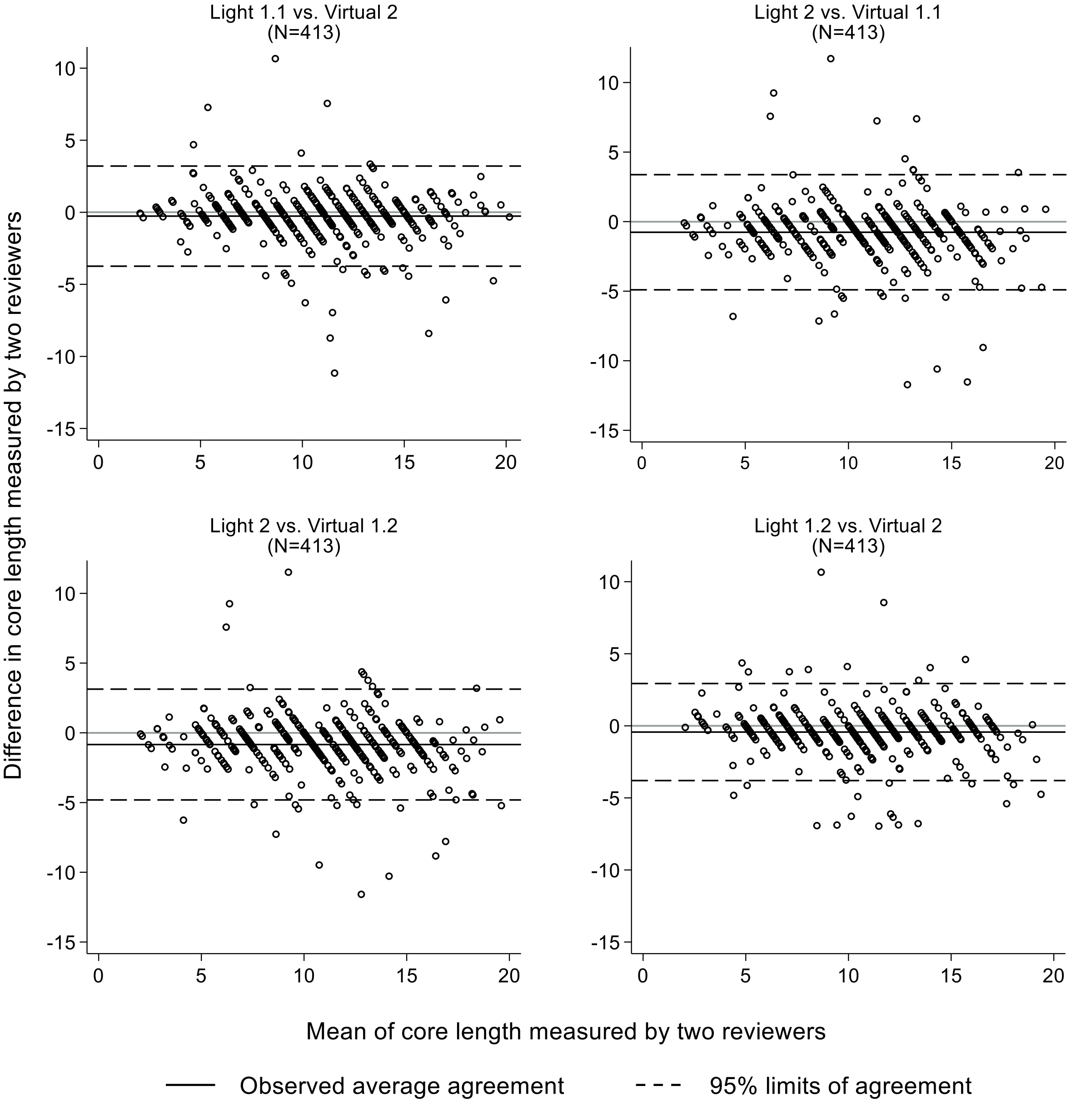

Supplementary Figure S17. Bland and Altman plot of the inter-method inter-observer agreement for the length of tumor in the biopsy core measured in millimeters, evaluated on the core level

Abbreviations: Light 1.1, First review by Reviewer 1 on light microscopy; Virtual 2, Reviewer 2 on virtual microscopy; Light 2, Reviewer 2 on light microscopy; Virtual 1.1, First review by Reviewer 1 on virtual microscopy; Virtual 1.2, Second review by Reviewer 1 on virtual microscopy; Light 1.2, Second review by Reviewer 1 on light microscopy

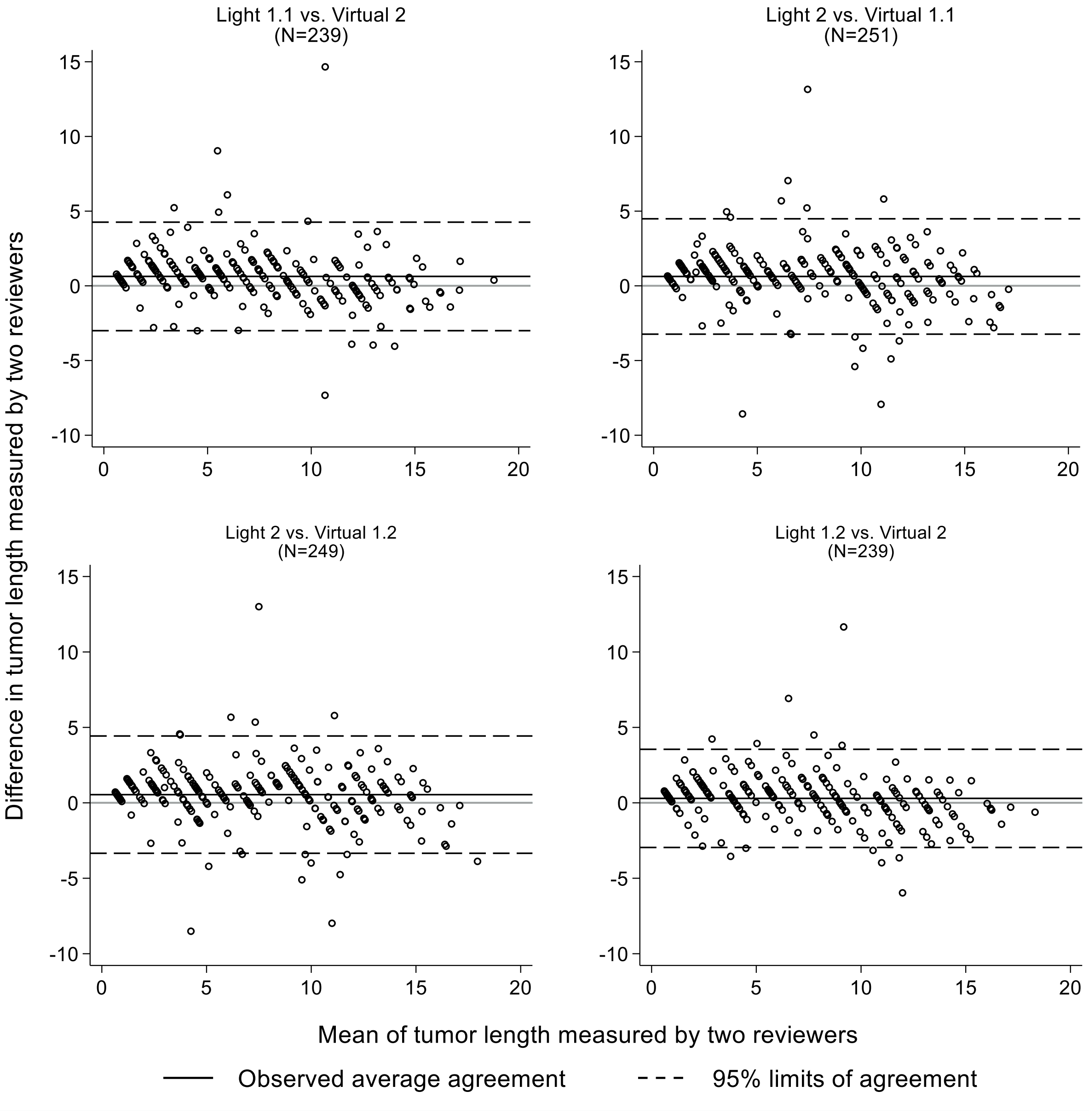

Supplementary Figure S18. Inter-method inter-observer agreement for characteristics evaluated on the core level

Abbreviations: Light 1.1, First review by Reviewer 1 on light microscopy; Virtual 2, Reviewer 2 on virtual microscopy; Light 2, Reviewer 2 on light microscopy; Virtual 1.1, First review by Reviewer 1 on virtual microscopy; Virtual 1.2, second review by Reviewer 1 on virtual microscopy; Light 1.2, second review by Reviewer 1 on light microscopy

Comparison	Sample size	Cohen's kappa (95% CI)
Primary Gleason pattern		
Light 1.1 vs. Virtual 2	243	0.76 (0.68, 0.83)
Light 2 vs. Virtual 1.1	251	0.79 (0.73, 0.86)
Light 2 vs. Virtual 1.2	250	0.80 (0.73, 0.86)
Light 1.2 vs. Virtual 2	243	0.88 (0.82, 0.94)
Secondary Gleason pattern		
Light 1.1 vs. Virtual 2	243	0.62 (0.54, 0.70)
Light 2 vs. Virtual 1.1	251	0.53 (0.45, 0.62)
Light 2 vs. Virtual 1.2	250	0.65 (0.57, 0.72)
Light 1.2 vs. Virtual 2	243	0.77 (0.71, 0.84)
Gleason score		
Light 1.1 vs. Virtual 2	243	0.80 (0.75, 0.85)
Light 2 vs. Virtual 1.1	251	0.76 (0.71, 0.81)
Light 2 vs. Virtual 1.2	250	0.79 (0.74, 0.84)
Light 1.2 vs. Virtual 2	243	0.87 (0.83, 0.91)
Gleason Grade Groups		
Light 1.1 vs. Virtual 2	243	0.82 (0.78, 0.86)
Light 2 vs. Virtual 1.1	251	0.81 (0.77, 0.85)
Light 2 vs. Virtual 1.2	250	0.82 (0.78, 0.87)
Light 1.2 vs. Virtual 2	243	0.88 (0.84, 0.91)
Cribriform pattern		
Light 1.1 vs. Virtual 2	243	0.51 (0.40, 0.62)
Light 2 vs. Virtual 1.1	251	0.48 (0.37, 0.59)
Light 2 vs. Virtual 1.2	250	0.46 (0.35, 0.57)
Light 1.2 vs. Virtual 2	243	0.54 (0.44, 0.65)
Poorly formed glands		
Light 1.1 vs. Virtual 2	243	0.57 (0.46, 0.68)
Light 2 vs. Virtual 1.1	251	0.49 (0.38, 0.60)
Light 2 vs. Virtual 1.2	250	0.53 (0.42, 0.64)
Light 1.2 vs. Virtual 2	243	0.66 (0.57, 0.76)
Comedonecrosis		
Light 1.1 vs. Virtual 2	243	0.62 (0.39, 0.85)
Light 2 vs. Virtual 1.1	251	0.68 (0.50, 0.86)
Light 2 vs. Virtual 1.2	250	0.66 (0.48, 0.84)
Light 1.2 vs. Virtual 2	243	0.59 (0.34, 0.83)
Perineural invasion		
Light 1.1 vs. Virtual 2	243	0.47 (0.33, 0.60)
Light 2 vs. Virtual 1.1	251	0.64 (0.52, 0.76)
Light 2 vs. Virtual 1.2	250	0.66 (0.55, 0.78)
Light 1.2 vs. Virtual 2	243	0.53 (0.38, 0.68)
Intraductal carcinoma		
Light 1.1 vs. Virtual 2	243	0.28 (-0.16, 0.72)
Light 2 vs. Virtual 1.1	251	0.36 (0.09, 0.62)
Light 2 vs. Virtual 1.2	250	0.31 (0.03, 0.59)
Light 1.2 vs. Virtual 2	243	0.59 (0.22, 0.96)
Mucinous carcinoma		
Light 1.1 vs. Virtual 2	243	0.24 (-0.17, 0.64)
Light 2 vs. Virtual 1.1	251	0.49 (0.21, 0.76)
Light 2 vs. Virtual 1.2	250	0.51 (0.24, 0.79)
Light 1.2 vs. Virtual 2	243	0.32 (-0.17, 0.82)

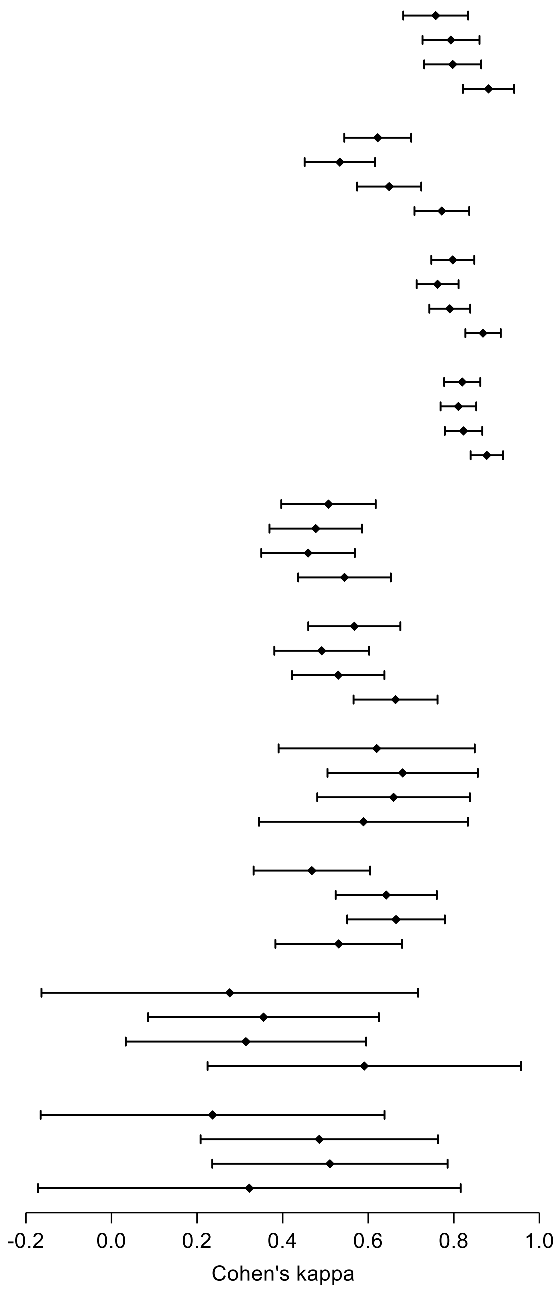

Supplementary Figure S19. Inter-method inter-observer agreement for characteristics evaluated on the slide level

Abbreviations: CI, confidence interval; Light 1.1, First review by Reviewer 1 on light microscopy; Virtual 2, Reviewer 2 on virtual microscopy; Light 2, Reviewer 2 on light microscopy; Virtual 1.1, First review by Reviewer 1 on virtual microscopy; Virtual 1.2, second review by Reviewer 1 on virtual microscopy; Light 1.2, second review by Reviewer 1 on light microscopy

Comparison	Sample size	Cohen's kappa (95% CI)
Acute inflammation		
Light 1.1 vs. Virtual 2	350	0.46 (0.27, 0.65)
Light 2 vs. Virtual 1.1	353	0.50 (0.32, 0.69)
Light 2 vs. Virtual 1.2	351	0.60 (0.43, 0.77)
Light 1.2 vs. Virtual 2	350	0.30 (0.11, 0.50)
Chronic inflammation		
Light 1.1 vs. Virtual 2	350	0.44 (0.34, 0.54)
Light 2 vs. Virtual 1.1	353	0.31 (0.24, 0.38)
Light 2 vs. Virtual 1.2	351	0.33 (0.25, 0.40)
Light 1.2 vs. Virtual 2	350	0.44 (0.35, 0.54)
High-grade prostatic intraepithelial neoplasia		
Light 1.1 vs. Virtual 2	350	0.16 (-0.02, 0.34)
Light 2 vs. Virtual 1.1	353	0.26 (0.09, 0.43)
Light 2 vs. Virtual 1.2	351	0.39 (0.20, 0.59)
Light 1.2 vs. Virtual 2	350	0.27 (0.06, 0.48)
Intraglandular inflamation		
Light 1.1 vs. Virtual 2	350	0.49 (0.31, 0.67)
Light 2 vs. Virtual 1.1	353	0.46 (0.31, 0.61)
Light 2 vs. Virtual 1.2	351	0.54 (0.37, 0.70)
Light 1.2 vs. Virtual 2	350	0.25 (0.08, 0.43)
Postatrophic hyperplasia		
Light 1.1 vs. Virtual 2	350	0.51 (0.25, 0.76)
Light 2 vs. Virtual 1.1	353	0.49 (0.27, 0.72)
Light 2 vs. Virtual 1.2	351	0.35 (0.11, 0.58)
Light 1.2 vs. Virtual 2	350	0.40 (0.12, 0.69)
Periglandular inflamation		
Light 1.1 vs. Virtual 2	350	0.37 (0.26, 0.48)
Light 2 vs. Virtual 1.1	353	0.18 (0.10, 0.26)
Light 2 vs. Virtual 1.2	351	0.18 (0.10, 0.27)
Light 1.2 vs. Virtual 2	350	0.37 (0.26, 0.49)
Stromal inflammation		
Light 1.1 vs. Virtual 2	350	0.37 (0.25, 0.48)
Light 2 vs. Virtual 1.1	353	0.30 (0.21, 0.39)
Light 2 vs. Virtual 1.2	351	0.32 (0.22, 0.41)
Light 1.2 vs. Virtual 2	350	0.38 (0.27, 0.48)

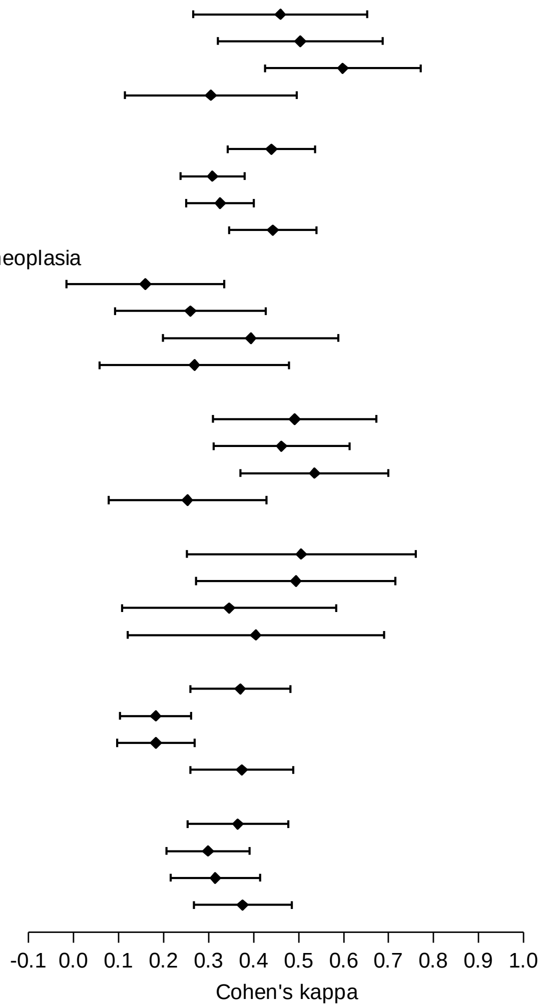

Supplementary Figure S20. Bland and Altman plot of the inter-method inter-observer agreement for the percentage of Gleason pattern 4, evaluated on the core level

Abbreviations: Light1.1, First review by Reviewer 1 on light microscopy; Virtual2, Reviewer 2 on virtual microscopy; Light2, Reviewer2 on light microscopy; Virtual1.1, First review by Reviewer 1 on virtual microscopy; Virtual1.2, second review by Reviewer1 on virtual microscopy; Light1.2, second review by Reviewer1 on light microscopy

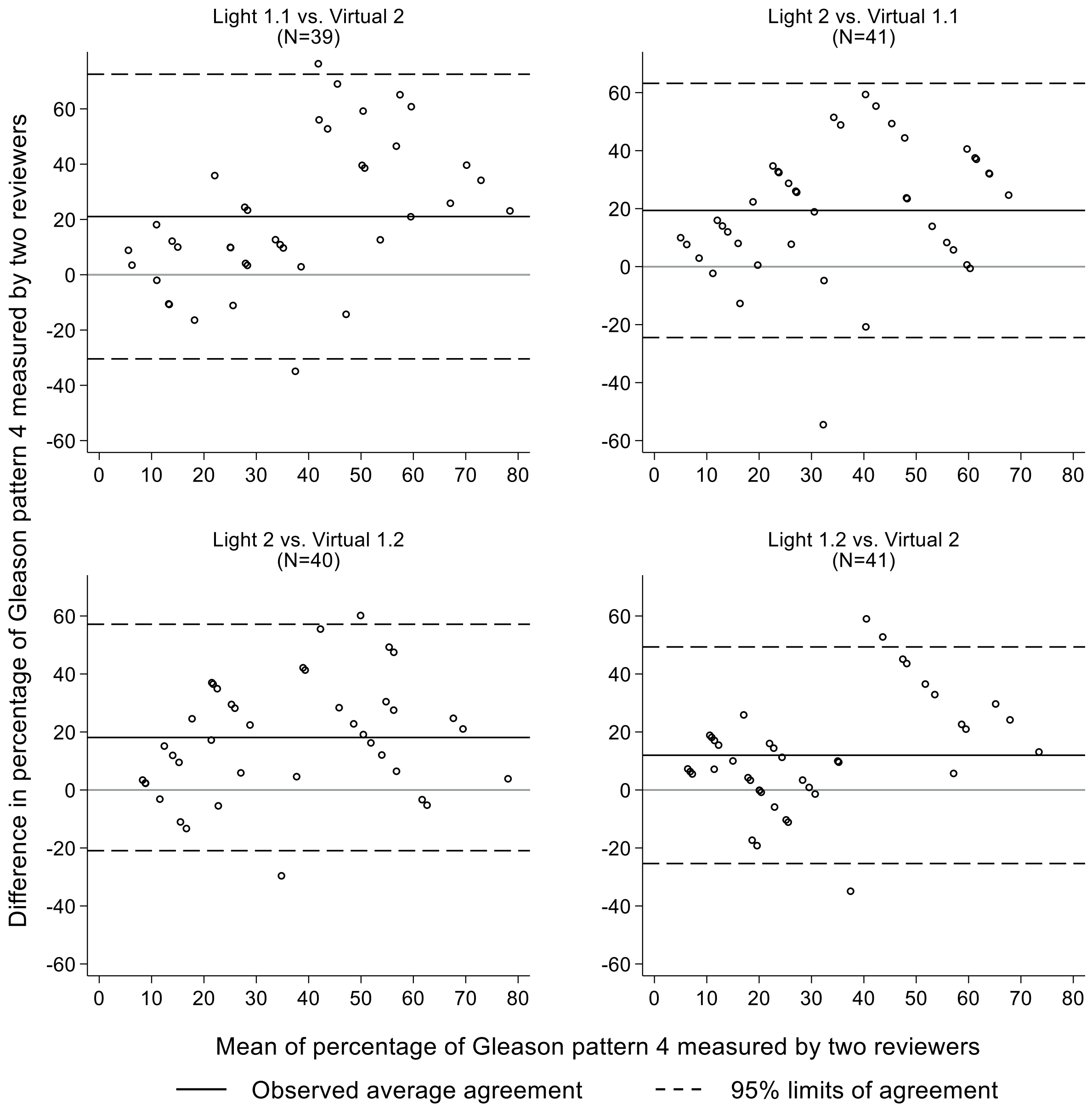

Supplementary Figure S21. Bland and Altman plot of the intra-method intra-observer agreement for the total biopsy length measured in millimeters, evaluated on the case level

Abbreviations: Light 1.1, First review by Reviewer 1 on light microscopy; Light 1.2, Second review by Reviewer 1 on light microscopy; Virtual 1.1, First review by Reviewer 1 on virtual microscopy; Virtual 1.2, Second review by Reviewer 1 on virtual microscopy

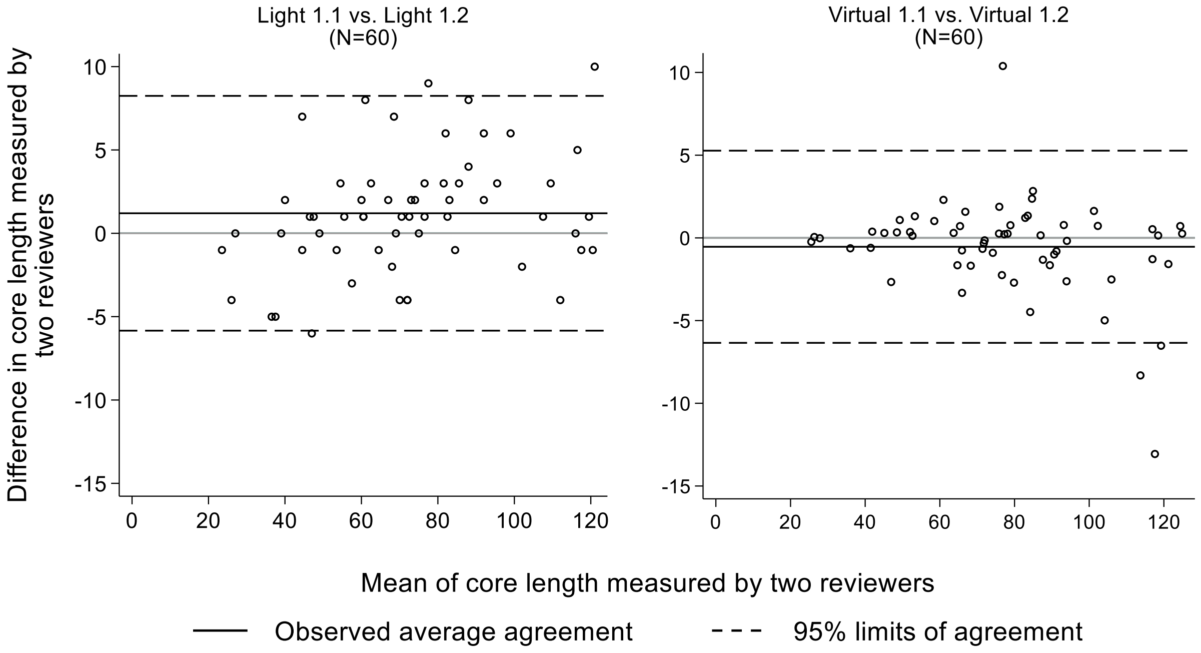

Supplementary Figure S22. Bland and Altman plot of the intra-method intra-observer agreement for the total length of tumor in the biopsy measured in millimeters, evaluated on the case level

Abbreviations: Light 1.1, First review by Reviewer 1 on light microscopy; Light 1.2, Second review by Reviewer 1 on light microscopy; Virtual 1.1, First review by Reviewer 1 on virtual microscopy; Virtual 1.2, Second review by Reviewer 1 on virtual microscopy

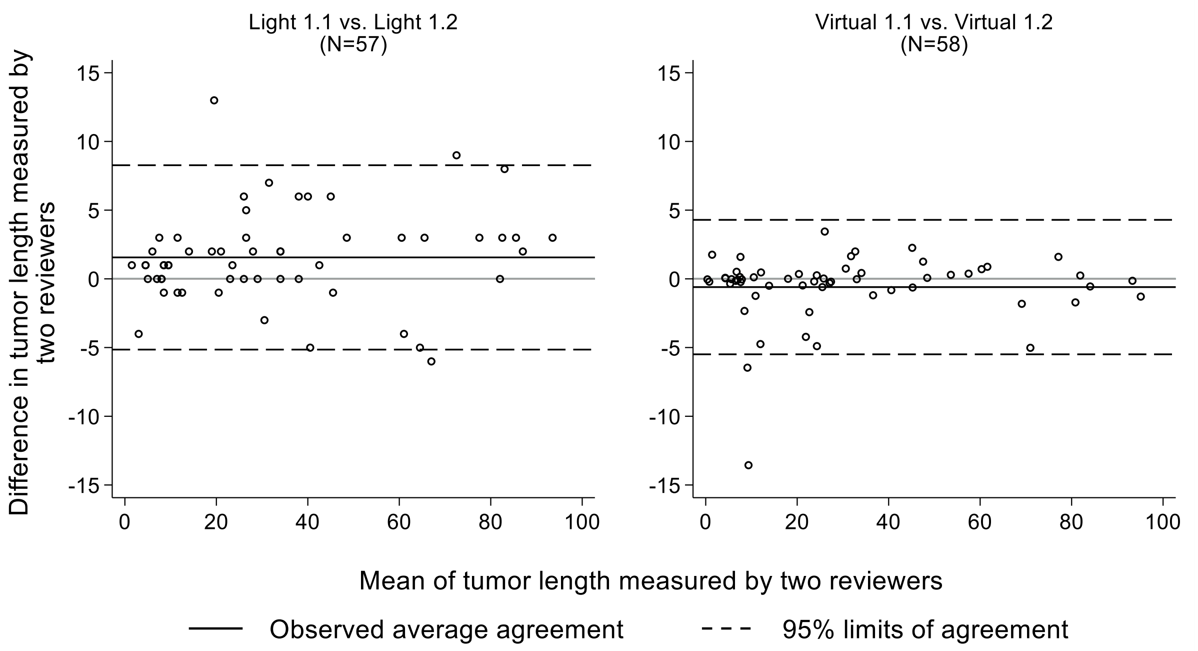

Supplementary Figure S23. Intra-method intra-observer agreement evaluated on the case level

Abbreviations: Light 1.1, First review by Reviewer 1 on light microscopy; Light 1.2, Second review by Reviewer 1 on light microscopy; Virtual 1.1, First review by Reviewer 1 on virtual microscopy; Virtual 1.2, Second review by Reviewer 1 on virtual microscopy

Comparison	Sample size	Cohen's kappa (95% CI)
Primary Gleason pattern		
Light 1.1 vs. Light 1.2	57	0.68 (0.50, 0.85)
Virtual 1.1 vs. Virtual 1.2	58	0.85 (0.72, 0.98)
Secondary Gleason pattern		
Light 1.1 vs. Light 1.2	57	0.69 (0.54, 0.84)
Virtual 1.1 vs. Virtual 1.2	58	0.82 (0.70, 0.94)
Gleason score		
Light 1.1 vs. Light 1.2	57	0.88 (0.81, 0.96)
Virtual 1.1 vs. Virtual 1.2	58	0.90 (0.83, 0.97)
Gleason Grade Groups		
Light 1.1 vs. Light 1.2	57	0.89 (0.82, 0.95)
Virtual 1.1 vs. Virtual 1.2	58	0.91 (0.84, 0.98)
Perineural invasion		
Light 1.1 vs. Light 1.2	57	0.69 (0.50, 0.89)
Virtual 1.1 vs. Virtual 1.2	58	0.64 (0.43, 0.86)

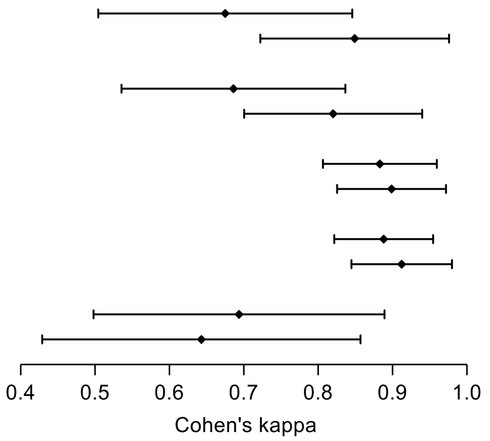

Supplementary Figure S24. Bland and Altman plot of the intra-method inter-observer agreement for the total biopsy length measured in millimeters, evaluated on the case level

Abbreviations: Light 1.1, First review by Reviewer 1 on light microscopy; Light 2, Reviewer 2 on light microscopy; Light 1.2, Second review by Reviewer 1 on light microscopy; Virtual 1.1, First review by Reviewer 1 on virtual microscopy; Virtual 2, Reviewer 2 on virtual microscopy; Virtual 1.2, Second review by Reviewer 1 on virtual microscopy

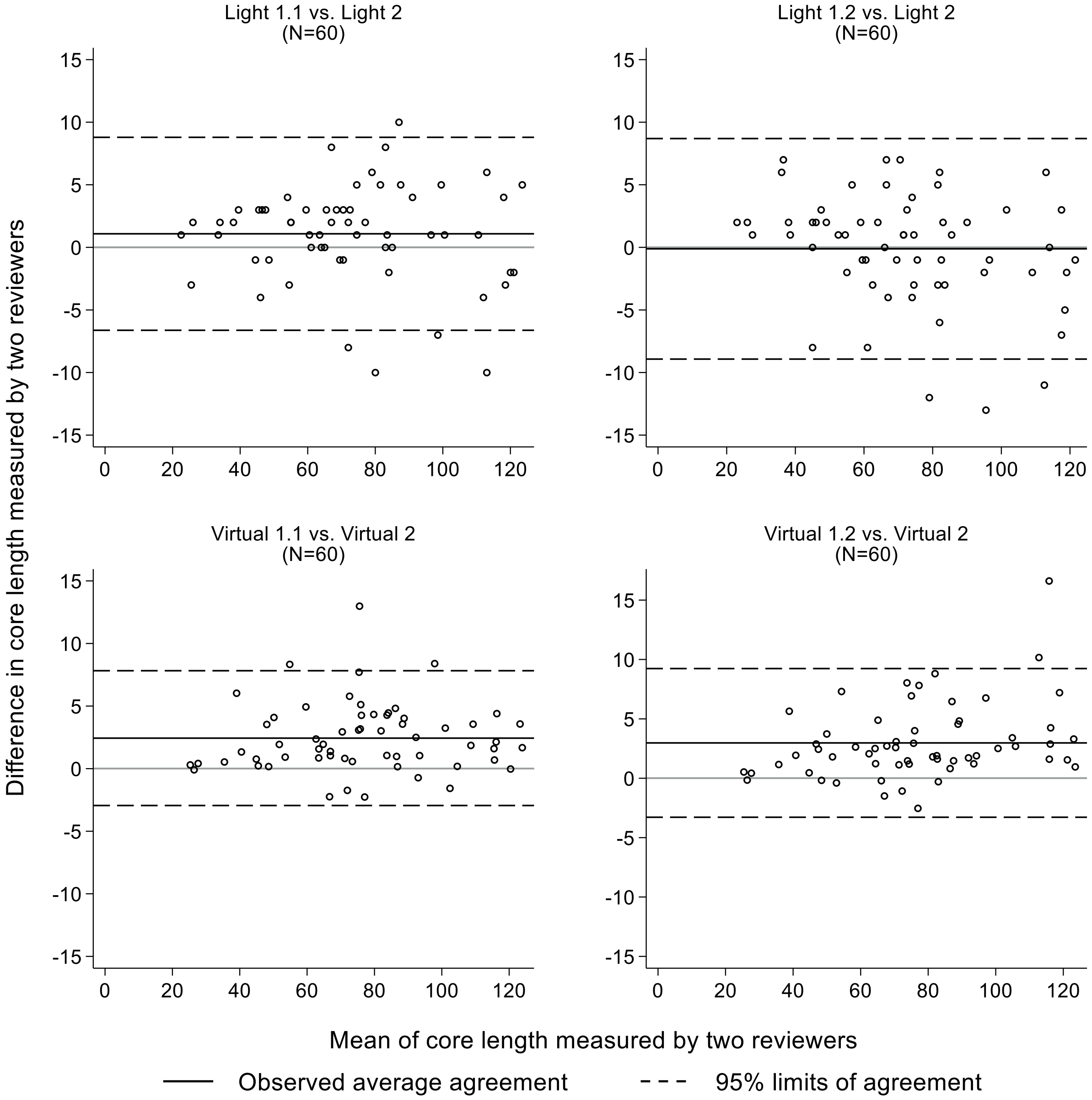

Supplementary Figure S25. Bland and Altman plot of the intra-method inter-observer agreement for the total length of tumor in the biopsy measured in millimeters evaluated on the case level

Abbreviations: Light 1.1, First review by Reviewer 1 on light microscopy; Light 2, Reviewer 2 on light microscopy; Light 1.2, Second review by Reviewer 1 on light microscopy; Virtual 1.1, First review by Reviewer 1 on virtual microscopy; Virtual 2, Reviewer 2 on virtual microscopy; Virtual 1.2, Second review by Reviewer 1 on virtual microscopy

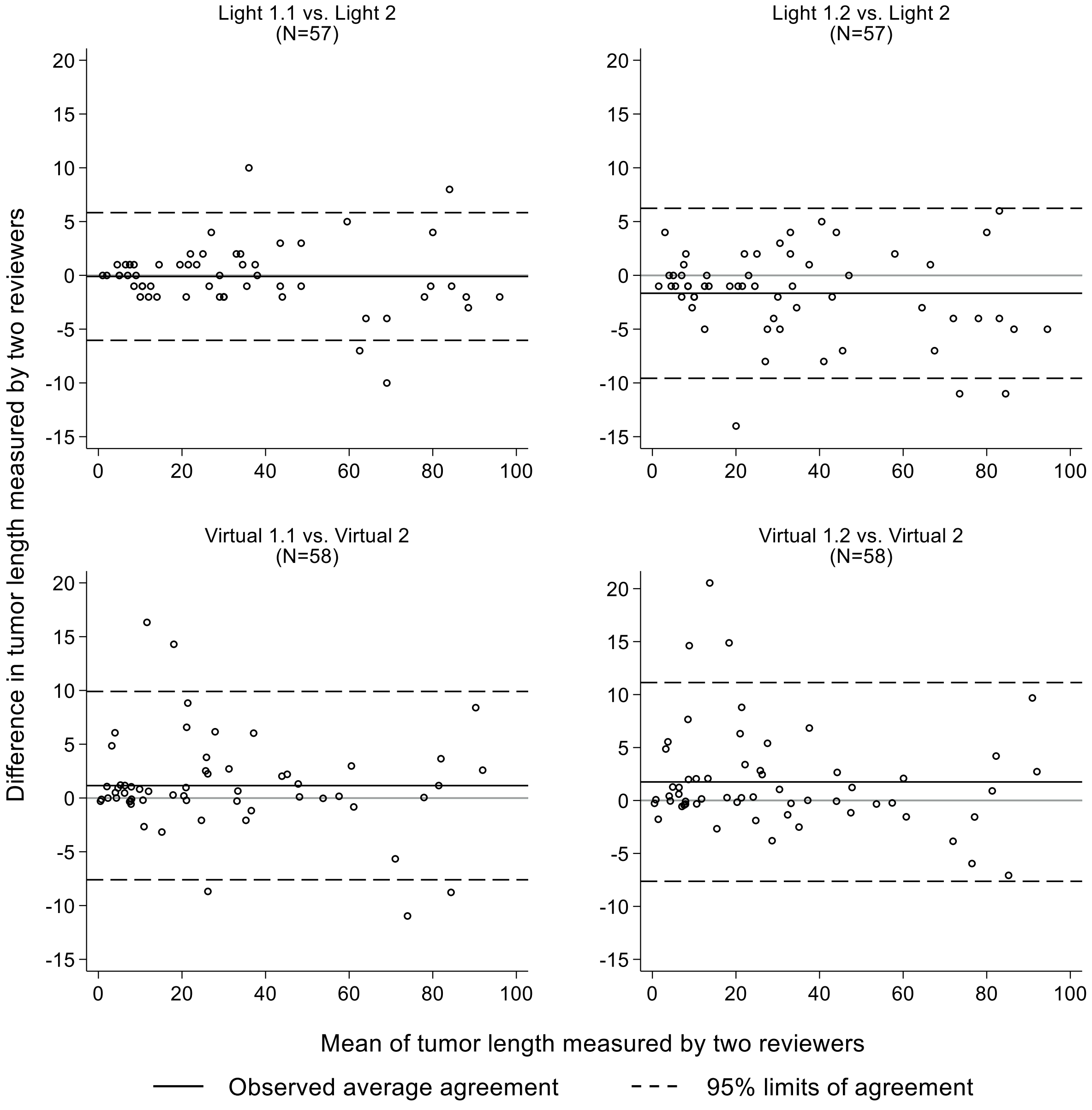

Supplementary Figure S26. Intra-method inter-observer agreement evaluated on the case level

Abbreviations: Light 1.1, First review by Reviewer 1 on light microscopy; Light 2, Reviewer 2 on light microscopy; Light 1.2, Second review by Reviewer 1 on light microscopy; Virtual 1.1, First review by Reviewer 1 on virtual microscopy; Virtual 2, Reviewer 2 on virtual microscopy; Virtual 1.2, Second review by Reviewer 1 on virtual microscopy

Comparison	Sample size	Cohen's kappa (95% CI)
Primary Gleason pattern		
Light 1.1 vs. Light 2	57	0.89 (0.79, 0.99)
Light 1.2 vs. Light 2	57	0.64 (0.47, 0.80)
Virtual 1.1 vs. Virtual 2	58	0.75 (0.59, 0.91)
Virtual 1.2 vs. Virtual 2	58	0.72 (0.56, 0.88)
Secondary Gleason pattern		
Light 1.1 vs. Light 2	57	0.83 (0.72, 0.95)
Light 1.2 vs. Light 2	57	0.66 (0.50, 0.82)
Virtual 1.1 vs. Virtual 2	58	0.72 (0.57, 0.86)
Virtual 1.2 vs. Virtual 2	58	0.67 (0.52, 0.82)
Gleason score		
Light 1.1 vs. Light 2	57	0.96 (0.91, 1.00)
Light 1.2 vs. Light 2	57	0.87 (0.79, 0.95)
Virtual 1.1 vs. Virtual 2	58	0.87 (0.79, 0.95)
Virtual 1.2 vs. Virtual 2	58	0.83 (0.73, 0.93)
Gleason Grade Groups		
Light 1.1 vs. Light 2	57	0.97 (0.93, 1.00)
Light 1.2 vs. Light 2	57	0.90 (0.83, 0.97)
Virtual 1.1 vs. Virtual 2	58	0.86 (0.78, 0.94)
Virtual 1.2 vs. Virtual 2	58	0.84 (0.74, 0.94)
Perineural invasion		
Light 1.1 vs. Light 2	57	0.89 (0.76, 1.00)
Light 1.2 vs. Light 2	57	0.73 (0.54, 0.92)
Virtual 1.1 vs. Virtual 2	58	0.40 (0.15, 0.66)
Virtual 1.2 vs. Virtual 2	58	0.61 (0.39, 0.83)

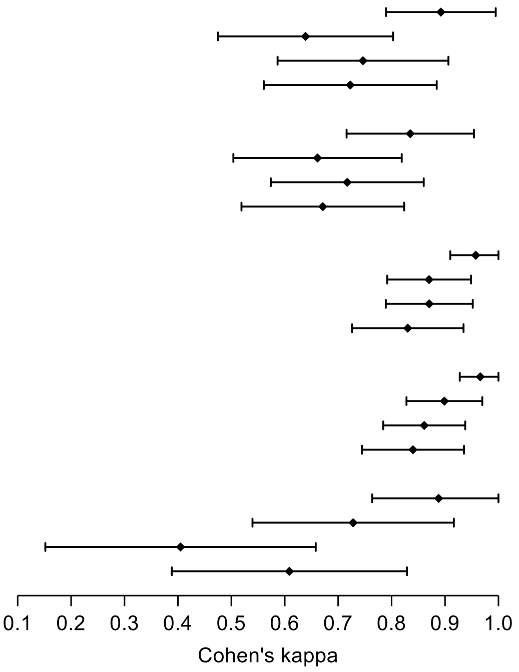

Supplementary Figure S27. Bland and Altman plot of the inter-method intra-observer agreement for the total biopsy length measured in millimeters, evaluated on the case level

Abbreviations: Light 1.1, First review by Reviewer 1 on light microscopy; Virtual 1.1, First review by Reviewer 1 on virtual microscopy; Virtual 1.2, Second review by Reviewer 1 on virtual microscopy; Light 2, Reviewer 2 on light microscopy; Virtual 2, Reviewer 2 on virtual microscopy; Light 1.2, Second review by Reviewer 1 on light microscopy

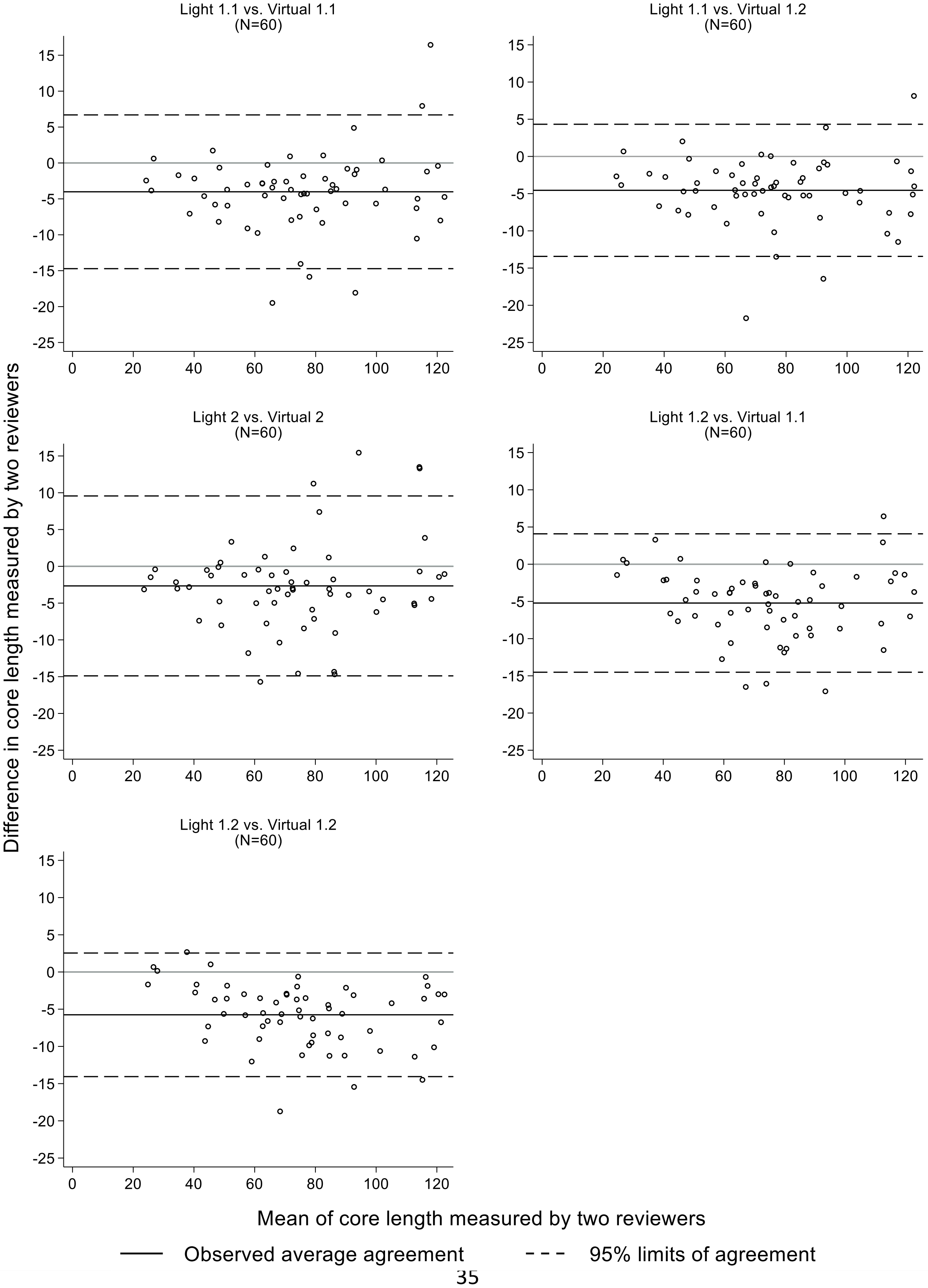

Supplementary Figure S28. Bland and Altman plot of the inter-method intra-observer agreement for the total length of tumor in the biopsy measured in millimeters, evaluated on the case level

Abbreviations: Light 1.1, First review by Reviewer 1 on light microscopy; Virtual 1.1, First review by Reviewer 1 on virtual microscopy; Virtual 1.2, Second review by Reviewer 1 on virtual microscopy; Light 2, Reviewer 2 on light microscopy; Virtual 2, Reviewer 2 on virtual microscopy; Light 1.2, Second review by Reviewer 1 on light microscopy

Note: Red lines show limits of agreement when outlier was removed from the analysis

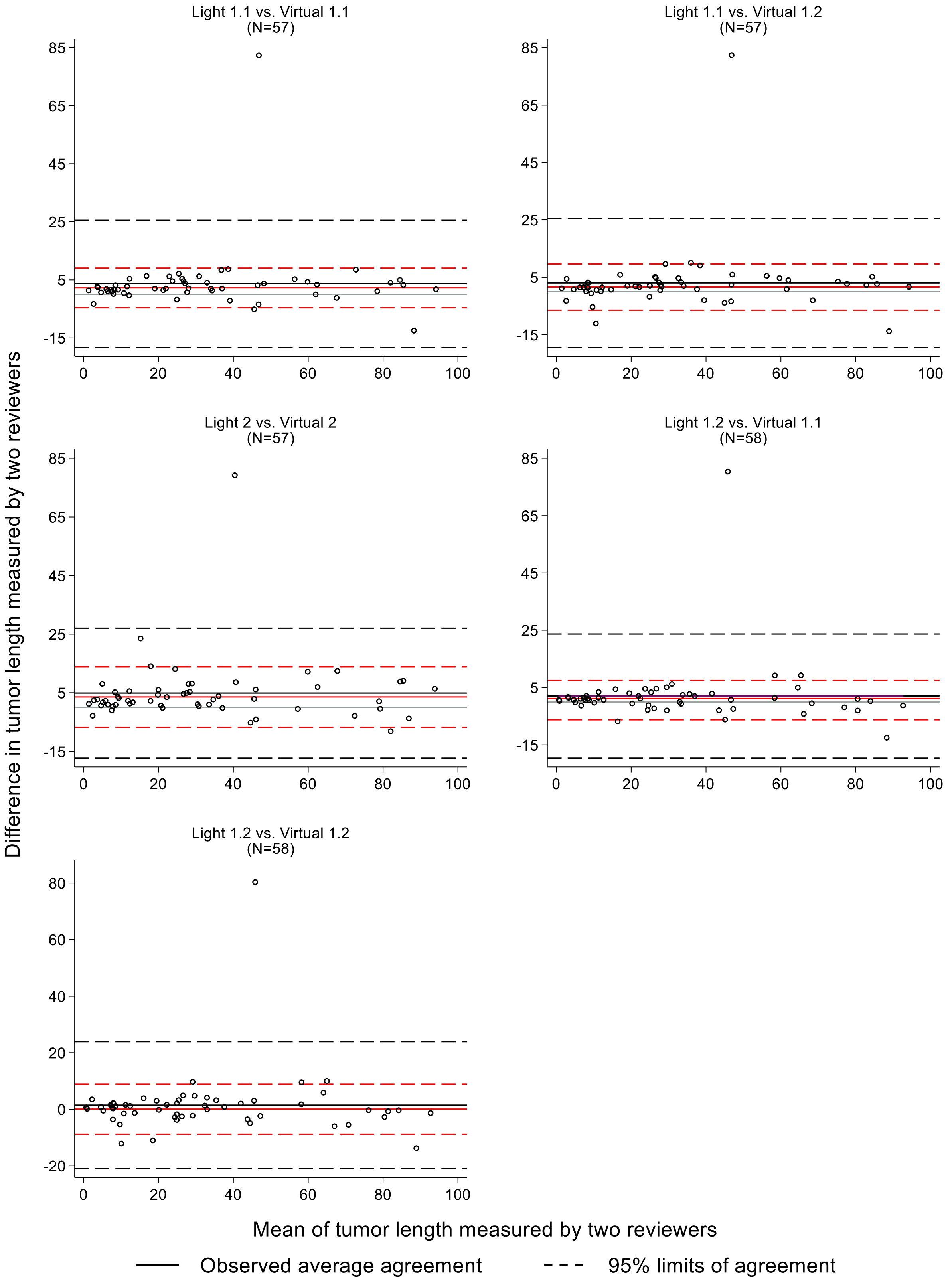

Supplementary Figure S29. Inter-method intra-observer agreement evaluated on the case level

Abbreviations: Light 1.1, First review by Reviewer 1 on light microscopy; Virtual 1.1, First review by Reviewer 1 on virtual microscopy; Virtual 1.2, Second review by Reviewer 1 on virtual microscopy; Light 2, Reviewer 2 on light microscopy; Virtual 2, Reviewer 2 on virtual microscopy; Light 1.2, Second review by Reviewer 1 on light microscopy

Comparison	Sample size	Cohen's kappa (95% CI)
Primary Gleason grade		
Light 1.1 vs. Virtual 1.1	57	0.85 (0.72, 0.98)
Light 1.1 vs. Virtual 1.2	57	0.77 (0.61, 0.92)
Light 2 vs. Virtual 2	57	0.63 (0.46, 0.80)
Light 1.2 vs. Virtual 1.1	58	0.69 (0.52, 0.87)
Light 1.2 vs. Virtual 1.2	58	0.67 (0.50, 0.85)
Secondary Gleason grade		
Light 1.1 vs. Virtual 1.1	57	0.77 (0.63, 0.90)
Light 1.1 vs. Virtual 1.2	57	0.86 (0.75, 0.97)
Light 2 vs. Virtual 2	57	0.55 (0.37, 0.72)
Light 1.2 vs. Virtual 1.1	58	0.70 (0.55, 0.84)
Light 1.2 vs. Virtual 1.2	58	0.69 (0.55, 0.84)
Gleason score		
Light 1.1 vs. Virtual 1.1	57	0.90 (0.81, 0.98)
Light 1.1 vs. Virtual 1.2	57	0.88 (0.80, 0.97)
Light 2 vs. Virtual 2	57	0.82 (0.71, 0.92)
Light 1.2 vs. Virtual 1.1	58	0.87 (0.78, 0.96)
Light 1.2 vs. Virtual 1.2	58	0.83 (0.72, 0.93)
Gleason Grade Groups		
Light 1.1 vs. Virtual 1.1	57	0.91 (0.83, 0.99)
Light 1.1 vs. Virtual 1.2	57	0.86 (0.76, 0.97)
Light 2 vs. Virtual 2	57	0.85 (0.75, 0.94)
Light 1.2 vs. Virtual 1.1	58	0.85 (0.75, 0.94)
Light 1.2 vs. Virtual 1.2	58	0.83 (0.72, 0.94)
Perineural invasion		
Light 1.1 vs. Virtual 1.1	57	0.69 (0.50, 0.89)
Light 1.1 vs. Virtual 1.2	57	0.73 (0.55, 0.92)
Light 2 vs. Virtual 2	57	0.54 (0.31, 0.77)
Light 1.2 vs. Virtual 1.1	58	0.52 (0.28, 0.76)
Light 1.2 vs. Virtual 1.2	58	0.72 (0.53, 0.92)

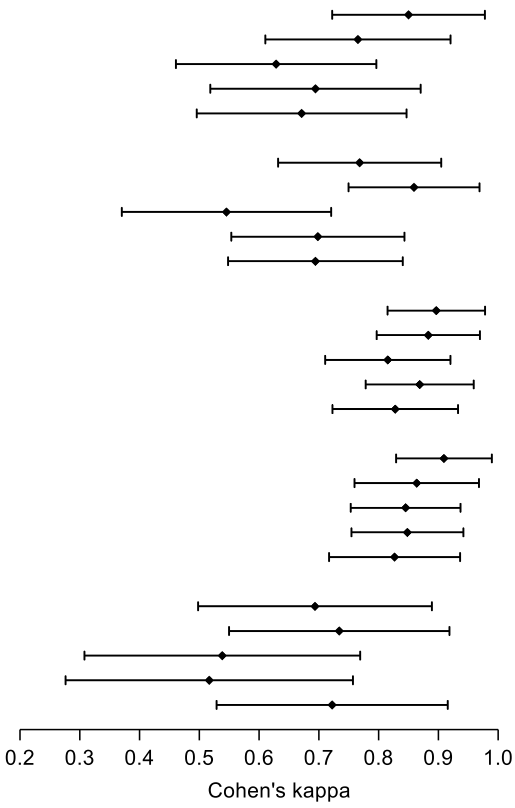

Supplementary Figure S30. Bland and Altman plot of the inter-method inter-observer agreement for the total biopsy length measured in millimeters, evaluated on the case level

Abbreviations: Light 1.1, First review by Reviewer 1 on light microscopy; Virtual 2, Reviewer 2 on virtual microscopy; Light 2, Reviewer 2 on light microscopy; Virtual 1.1, First review by Reviewer 1 on virtual microscopy; Virtual 1.2, second review by Reviewer 1 on virtual microscopy; Light 1.2, second review by Reviewer 1 on light microscopy

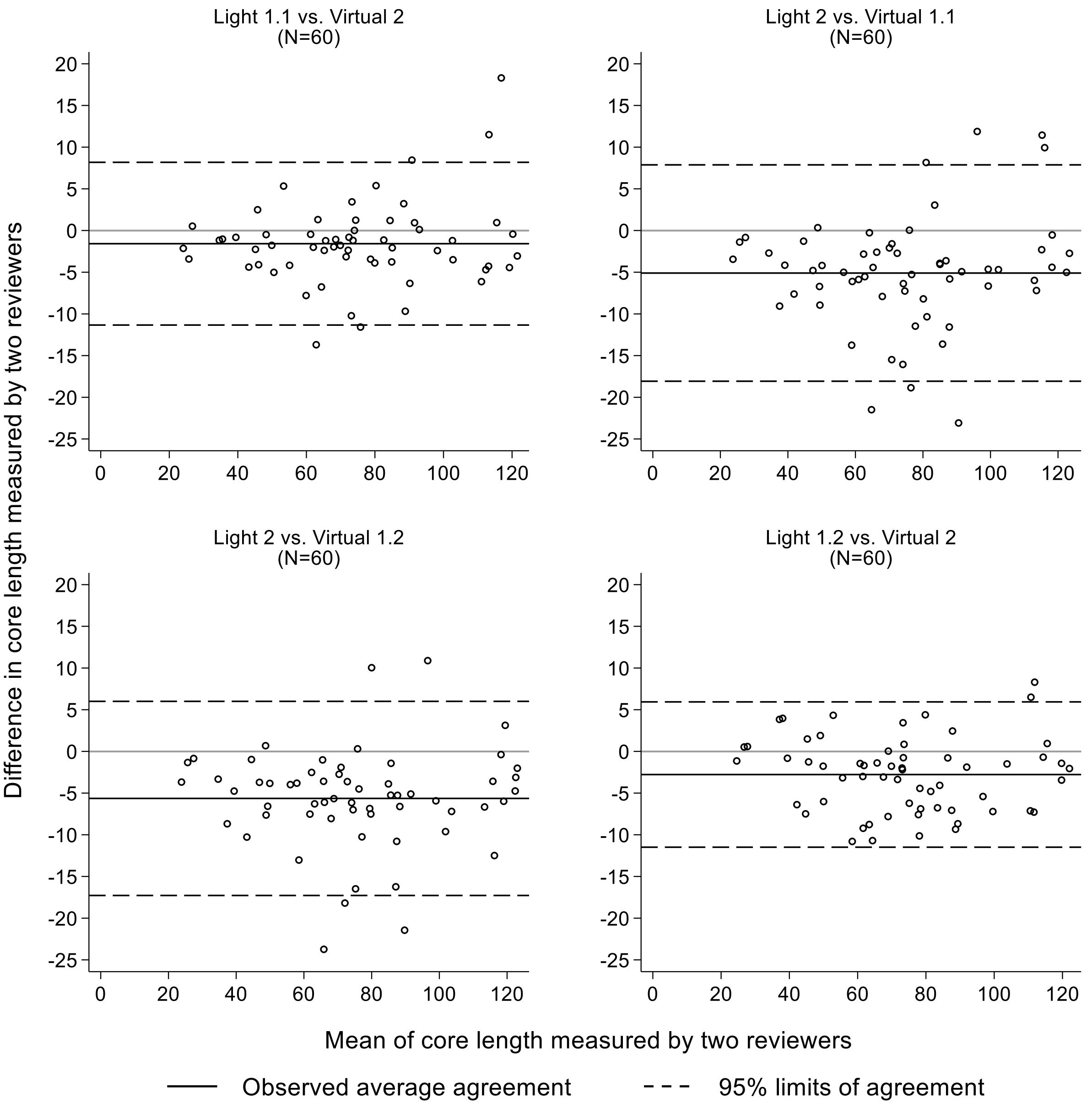

Supplementary Figure S31. Bland and Altman plot of the inter-method inter-observer agreement for the total length of tumor in the biopsy measured in millimeters, evaluated on the case level

Abbreviations: Light 1.1, First review by Reviewer 1 on light microscopy; Virtual 2, Reviewer 2 on virtual microscopy; Light 2, Reviewer 2 on light microscopy; Virtual 1.1, First review by Reviewer 1 on virtual microscopy; Virtual 1.2, second review by Reviewer 1 on virtual microscopy; Light 1.2, second review by Reviewer 1 on light microscopy

Note: Red lines show limits of agreement when outlier was removed from the analysis

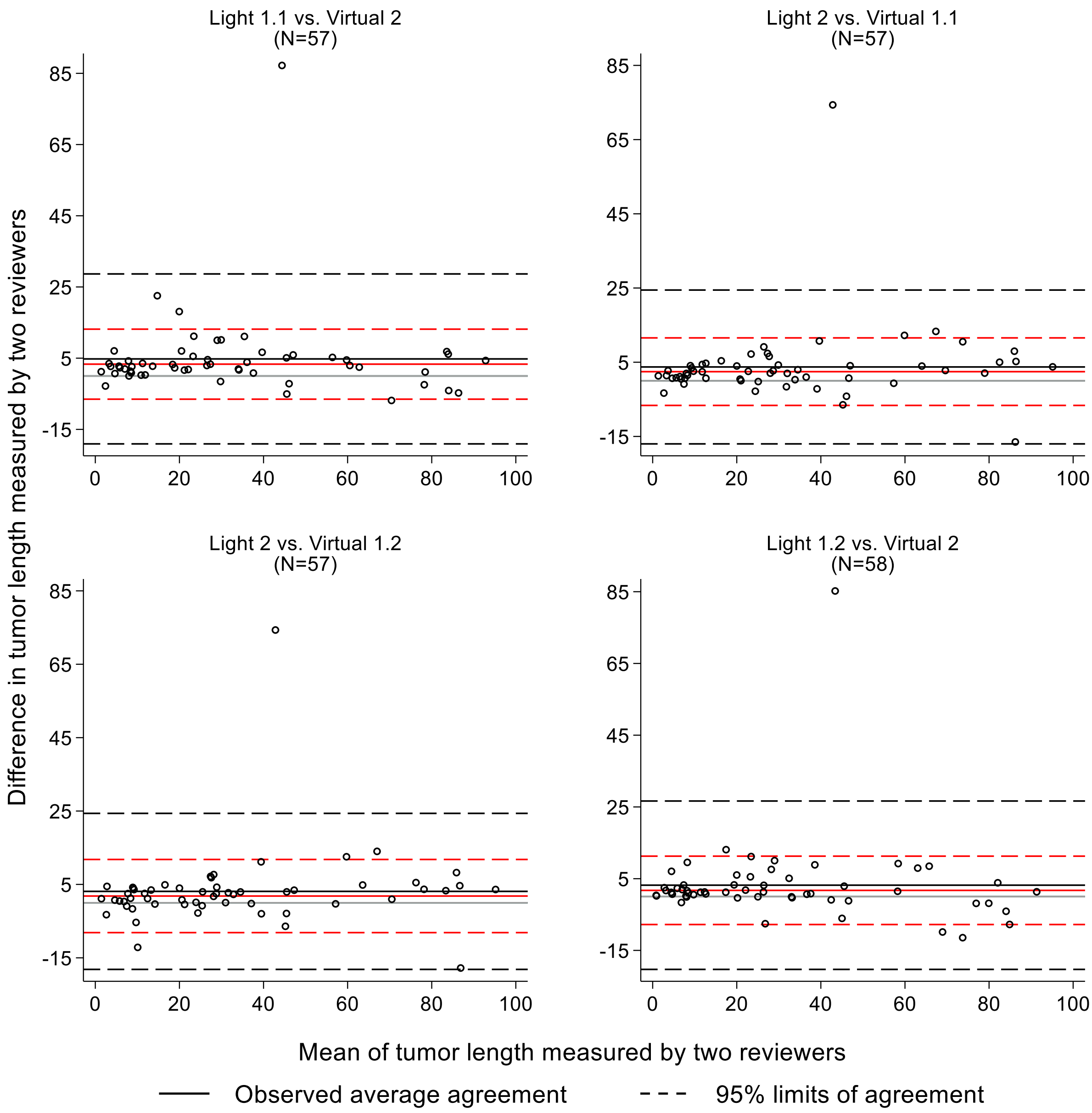

Supplementary Figure S32. Inter-method inter-observer agreement evaluated on the case level

Abbreviations: Light 1.1, First review by Reviewer 1 on light microscopy; Virtual 2, Reviewer 2 on virtual microscopy; Light 2, Reviewer 2 on light microscopy; Virtual 1.1, First review by Reviewer 1 on virtual microscopy; Virtual 1.2, second review by Reviewer 1 on virtual microscopy; Light 1.2, second review by Reviewer 1 on light microscopy

Comparison	Sample size	Cohen's kappa (95% CI)
Primary Gleason grade		
Light 1.1 vs. Virtual 2	57	0.66 (0.49, 0.84)
Light 2 vs. Virtual 1.1	57	0.80 (0.66, 0.94)
Light 2 vs. Virtual 1.2	57	0.72 (0.56, 0.88)
Light 1.2 vs. Virtual 2	58	0.82 (0.68, 0.97)
Secondary Gleason grade		
Light 1.1 vs. Virtual 2	57	0.62 (0.45, 0.78)
Light 2 vs. Virtual 1.1	57	0.65 (0.48, 0.81)
Light 2 vs. Virtual 1.2	57	0.74 (0.60, 0.88)
Light 1.2 vs. Virtual 2	58	0.85 (0.75, 0.96)
Gleason score		
Light 1.1 vs. Virtual 2	57	0.83 (0.72, 0.93)
Light 2 vs. Virtual 1.1	57	0.85 (0.76, 0.94)
Light 2 vs. Virtual 1.2	57	0.87 (0.78, 0.96)
Light 1.2 vs. Virtual 2	58	0.94 (0.88, 1.00)
Gleason Grade Groups		
Light 1.1 vs. Virtual 2	57	0.83 (0.74, 0.93)
Light 2 vs. Virtual 1.1	57	0.90 (0.81, 0.98)
Light 2 vs. Virtual 1.2	57	0.87 (0.78, 0.97)
Light 1.2 vs. Virtual 2	58	0.93 (0.85, 1.00)
Perineural invasion		
Light 1.1 vs. Virtual 2	57	0.58 (0.36, 0.80)
Light 2 vs. Virtual 1.1	57	0.65 (0.44, 0.86)
Light 2 vs. Virtual 1.2	57	0.69 (0.49, 0.89)
Light 1.2 vs. Virtual 2	58	0.64 (0.43, 0.86)

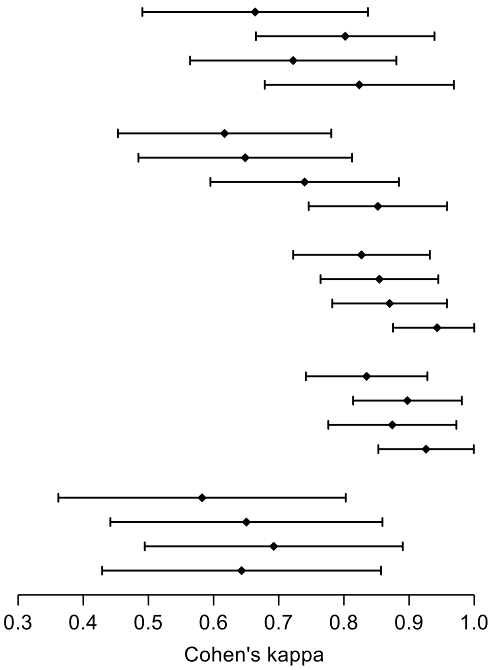

Supplement: Supplementary file 1 — Supplementary Information. [file 41598_2021_82911_MOESM1_ESM.pdf]
